# Shape Similarity, Better than Semantic Membership, Accounts for the Structure of Visual Object Representations in a Population of Monkey Inferotemporal Neurons

**DOI:** 10.1371/journal.pcbi.1003167

**Published:** 2013-08-08

**Authors:** Carlo Baldassi, Alireza Alemi-Neissi, Marino Pagan, James J. DiCarlo, Riccardo Zecchina, Davide Zoccolan

**Affiliations:** 1 Department of Applied Science and Technology & Center for Computational Sciences, Politecnico di Torino, Torino, Italy; 2 Human Genetics Foundation (HuGeF), Torino, Torino, Italy; 3 International School for Advanced Studies (SISSA), Trieste, Italy; 4 Department of Brain and Cognitive Sciences and McGovern Institute for Brain Research, Massachusetts Institute of Technology (MIT), Cambridge, Massachusetts, United States of America; 5 Department of Psychology, University of Pennsylvania, Philadelphia, Pennsylvania, United States of America; Philipps-University Marburg, Germany

## Abstract

The anterior inferotemporal cortex (IT) is the highest stage along the hierarchy of visual areas that, in primates, processes visual objects. Although several lines of evidence suggest that IT primarily represents visual shape information, some recent studies have argued that neuronal ensembles in IT code the semantic membership of visual objects (i.e., represent conceptual classes such as animate and inanimate objects). In this study, we investigated to what extent semantic, rather than purely visual information, is represented in IT by performing a multivariate analysis of IT responses to a set of visual objects. By relying on a variety of machine-learning approaches (including a cutting-edge clustering algorithm that has been recently developed in the domain of statistical physics), we found that, in most instances, IT representation of visual objects is accounted for by their similarity at the level of shape or, more surprisingly, low-level visual properties. Only in a few cases we observed IT representations of semantic classes that were not explainable by the visual similarity of their members. Overall, these findings reassert the primary function of IT as a conveyor of explicit visual shape information, and reveal that low-level visual properties are represented in IT to a greater extent than previously appreciated. In addition, our work demonstrates how combining a variety of state-of-the-art multivariate approaches, and carefully estimating the contribution of shape similarity to the representation of object categories, can substantially advance our understanding of neuronal coding of visual objects in cortex.

## Author Summary

To build meaningful representations of the external word, the stream of sensory information that reaches our senses is continuously processed and interpreted by the brain. Ultimately, such a processing allows the brain to arrange sensory (e.g., visual) inputs into a hierarchy of categories (such as animate and inanimate objects) and sub-categories (such as faces, animals, buildings, tools, etc). Crucially, while many objects can be assigned to the same category based on their visual similarity (e.g., oranges and apples), formation of most categories also requires arbitrarily associating objects sharing similar functions/meaning, but not similar shape (e.g., bananas and apples). A long-standing debate exists about whether the representation of visual objects in the higher visual centers of the brain (such as the inferotemporal cortex; IT) purely reflects shape similarity or also (and, perhaps, mainly) shape-unrelated categorical knowledge. In this study, we have addressed this issue by applying a variety of computational approaches. Our results show that the response patterns of a population of inferotemporal neurons are better accounted for by shape similarity than categorical membership. This reasserts the primary function of IT as a visual area and demonstrates how state-of-the-art computational approaches can advance our understanding of neuronal coding in the brain.

## Introduction

In primates, visual object information is processed through a hierarchy of cortico-cortical stages (the *ventral visual pathway*) that culminates with the inferotemporal cortex (IT) [1]–[6]. Uncovering the nature of visual object representations in IT is central to our understanding of how visually presented objects are perceived, identified and categorized, yet it is extremely challenging. In fact, because of the non-linear mapping between the visual input space and IT neuronal responses, it is virtually impossible to precisely estimate the tuning of individual IT neurons over the image space (but see [7], [8]). As a result, it is somewhat arbitrary to assign IT the proper rank along the continuum that goes from extraction of simple visual features to formation of conceptual, semantic categories – are IT neurons closer to the local edge detectors found in primary visual areas or to the concept cells recently found in human middle temporal lobe [9]–[11]?

While most literature supports the notion that IT neurons code moderately to highly complex configurations of visual features [2], [6], [12], [13], recent work has argued that IT neuronal ensembles code the semantic membership of visual objects (i.e., represent behaviorally salient conceptual categories, such as animate and non-animate objects, animals, body parts, etc) rather than their visual properties [14]. A related study also compared how a set of visual objects was represented in monkey IT and its human homologous, finding that many semantic categories were represented equally well in both species (with a primary, sharp distinction between animate and inanimate objects) and reporting the inadequacy of various image-based similarity metrics to account for the observed patterns of neuronal responses [15]. Finally, a recent fMRI study concluded that object representations in monkey IT are spatially segregated according to semantic relationship [16], a finding that matches the segregation by function/meaning (rather than by shape) found in the topography of human high-level representations of visual objects [17]–[24].

Finding that abstract category information is represented in IT is not surprising per se, since several studies have shown how IT neurons can represent the association of arbitrary image pairs, either through explicit [25], [26] or implicit [27]–[29] associative learning. However, while these mechanisms can explain why extensively trained categories [30] or behaviorally salient categories (such as faces and body parts [31]–[34]) are represented in IT, they can hardly explain why category information was found to be represented in IT more systematically and robustly than visual shape information [14], [15]. In fact, several studies have shown that IT neurons are robustly tuned for object-defining visual features [7], [8], [35]–[39] and one study has shown that in IT, differently from prefrontal cortex, semantic category information is not greater than what expected based on the visual similarity of category members [40]. Finally, a very recent monkey fMRI study has found no sharp segregation between the representations of animate and inanimate objects in IT [34].

In this study, we have applied an array of multivariate approaches (some of which were recently developed in the domain of statistical mechanics) to investigate how an IT neuronal population represents pictures of natural objects. Our analysis shows that neuronal representations in IT largely depend on objects' similarity at the level of shape or, more surprisingly, low-level visual properties, with semantic membership only accounting for the representation of a few, behaviorally salient categories of animate objects (such as four-limbed animals and birds). Overall, these findings show that monkey IT is primarily a conveyor of explicit visual shape information, in which a surprisingly broad spectrum of visual feature complexity is represented.

## Results

**Figure 1:**
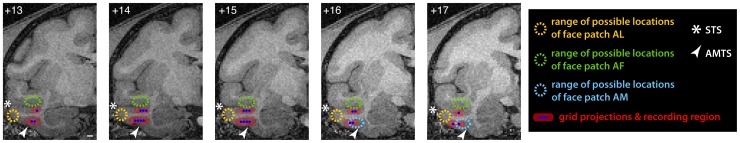
Recording locations. The blue dots show the projections of the recording chamber grid-point locations from the top of the skull to the ventral bank of the superior temporal sulcus (STS) and the ventral surface lateral to the anterior middle temporal sulcus (AMTS). The projections are shown over a sequence of MRI images (spanning a 13–17 anteroposterior range; Horsley-Clarke coordinates) that were collected, for one of the monkeys, before the chamber implant surgery. Only the grid locations in which the electrode was inserted at least once are shown. The red-shaded areas highlight the estimated cortical span that was likely sampled during recording, given that: 1) each electrode penetration usually spanned the whole depth of the targeted cortical bank (either STS or AMTS); and 2) the upper bound of the variability of each recording location along the mediolateral axis (due to bending of the electrode during insertion) can be estimated as ±2 mm [80]. The figure also shows the range of possible locations of the three anterior face patches (AL, AF and AM) according to [33], so as to highlight their potential overlap with the recording locations.

In this study, we recorded 94 well-isolated single units from the anterior inferotemporal cortex (IT) of two monkeys. Neurons were sampled across a ∼5×4 mm area of the ventral superior temporal sulcus (STS) and ventral surface lateral to the anterior middle temporal sulcus (AMTS), as shown in Figure 1 (see blue dots and red-shaded areas). No attempt was done to target specific IT patches containing cells with similar preference for faces, such as the AF (anterior fundus), AL (anterior lateral) and AM (anterior medial) face patches [31]–[33] (the range of possible locations of these patches is also shown in Fig. 1, based on [33]), or other IT regions that are rich of face selective neurons (summarized in [41], [42]). Finally, for a better comparison with previous findings, it is important to notice that we recorded from a region with a smaller anteroposterior (AP) and mediolateral (ML) extent than the region sampled by [14], although both regions were roughly centered at the same AP position in anterior IT (compare Fig. 1 with Fig. 1 in [14]).

**Figure 2:**
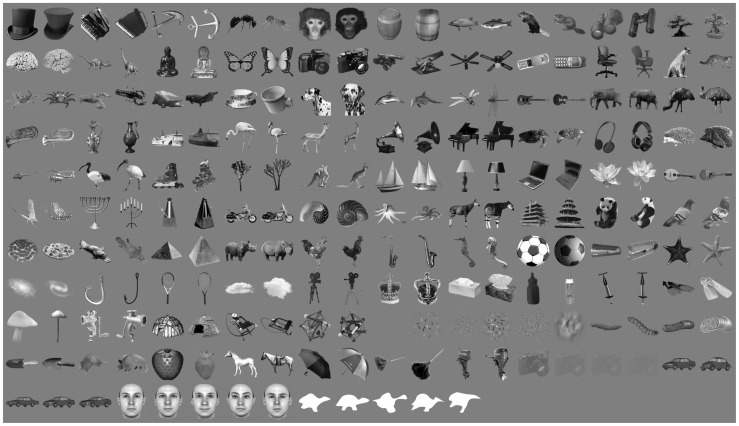
The stimulus set. The full set of 213 objects used in our study. The set consists of: i) 188 images of real-world objects belonging to 94 different categories (e.g., two hats, two accordions, two monkey faces, etc.); ii) 5 cars, 5 human faces, and 5 abstract silhouettes; iii) 5 patches of texture (e.g., random dots and oriented bars); iv) a blank frame; v) 4 low contrast (10%, 3%, 2% and 1.5%) images of one of the objects (a camera).

All neurons were probed with a set of 213 grayscale pictures of natural objects (see Fig. 2) presented at a rate of 5 images/s, while the animals were engaged in a simple object detection task. To understand how these objects were mapped into the IT neuronal space, we used linear classifiers and a variety of clustering algorithms, and we measured to what extent object clusters in the IT neuronal representation could be accounted by three different object attributes: 1) shared semantic membership; 2) shared shape features (i.e., shape similarity); and 3) shared low-level visual properties.

### Gradient in object area explains object clustering at the most “superordinate” level

**Figure 3:**
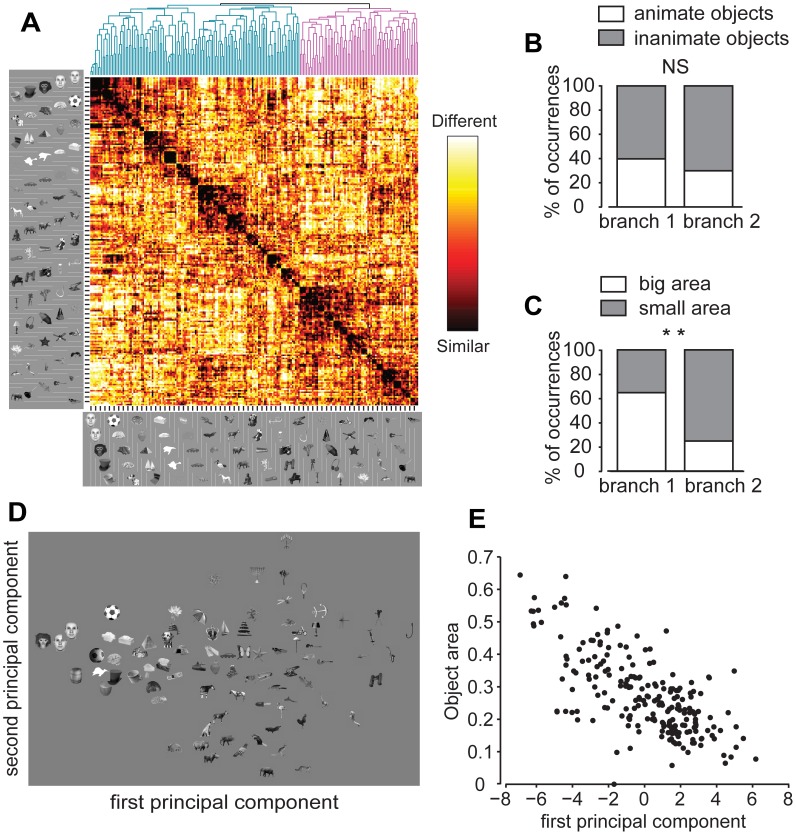
Similarity matrix, hierarchical clustering and PCA of IT population responses to visual objects. (A) Each pixel in the matrix color-codes the correlation (i.e., similarity) between the neuronal population vectors representing a pair of visual objects. The order of the objects along the axes is defined by the dendrogram produced by hierarchical clustering of the population vectors (to avoid crowding, one every three objects is shown; the complete object set is shown in Fig. 2). The first two branches of the dendrogram (shown at the top) are colored in cyan and magenta. (B) The fraction of animate and inanimate objects is not significantly different in the first two branches of the dendrogram (NS, *p*>0.1, *χ* ^2^ test). (C) The proportion of large and small objects is significantly different in the first two branches of the dendrogram (**, *p*<0.001, *χ* ^2^ test), (D) Layout of visual objects in the two-dimensional space defined by the first two principal components of the IT population responses (to avoid crowding, only some of the objects are shown). (E) Object area and object ranking along the first principal component are linearly related (*r* = −0.69, *p*<0.001, *t*-test).

The nature of visual object representations in IT can be studied by examining what features are shared by objects that produce similar population responses in the IT neuronal representation space. The similarity between the neuronal representations of a pair of visual objects (*neuronal-level similarity* in the following) was computed as the Pearson correlation coefficient of the normalized population response vectors produced by the two objects (see Materials and Methods). To gain some intuition into possible trends in the representation of our object set, the neuronal-level similarity between each object pair was color-coded in the matrix shown in Figure 3A. The order of the objects along the axes of the similarity matrix was determined by the dendrogram shown at the top, which was obtained by applying an agglomerative hierarchical clustering algorithm to the neural population vectors. This allowed objects evoking similar population responses to lie nearby in the matrix, so that clusters of objects that were similar in the neural representation space appeared as compact dark squares along the diagonal of the matrix.

Contrary to what was recently reported by [14], [15], but in agreement with [34], visual inspection of the similarity matrix revealed that objects did not show a tendency to cluster into two main, compact clusters, corresponding to the semantic categories of animate and inanimate entities. For instance, the set of faces (top of the ordinate matrix axis) was separated from the set of birds and four-limbed animals (approximately in the middle of the axis) by many inanimate objects. Other animate objects (such as insects, crustaceans, fishes, and some four-limbed animals) were scattered all over the matrix axis and intermixed with inanimate objects (such as man-made tools, trees, flowers, etc). Finally, although some animate objects, such as faces, appeared to cluster according to the subordinate semantic category they belonged to (i.e., the face category), such clusters were generally embedded within larger clusters of animate and inanimate objects with similar shape (i.e., the face cluster lay within a larger group of similarly round shapes – a cup, a ball, a brain, an urchin, etc.). To better quantify whether a segregation between animate and inanimate objects could be observed at the top level of the dendrogram obtained by hierarchical clustering, we measured the fraction of animate and inanimate objects in the first two branches of the dendrogram (i.e., the cyan vs. the magenta branch in the dendrogram shown in Fig. 3A). Animate objects amounted to ∼40% of the total in both branches and their fraction did not significantly differ in the two branches (*p*>0.1, *χ* ^2^ test; see Fig. 3B).

While animate and inanimate objects were not sharply segregated in the neuronal representation space, a different property appeared to determine object clustering in the two top-level branches of the dendrogram – a gradient in object area could be observed along the matrix axes, with bulkier objects (e.g., faces and other round shapes) at one end of the axes and thinner objects (e.g., an ant, a dolphin, a guitar, etc) at the other end. To quantify this trend, objects were divided in two equally sized subsets of “large” and “small” objects, depending on whether their area was above or below the median of the full object set (object area is defined in Materials and Methods). The proportion of large and small objects was significantly different in the first two branches of the dendrogram (*p*<0.001, *χ* ^2^ test), with large objects representing more than 60% of the total in one branch and only about 20% in the other (Fig. 3C).

To further investigate what properties shaped the representation of the objects in the IT neuronal space, we performed a Principal Component Analysis (PCA) of the recorded neuronal population vectors. The total variance explained by the first two principal components was fairly low (∼15%). This is not surprising, since our object set was highly varied in terms of visual properties and shape features and it is unlikely that high-level visual neurons, such as those sampled in our IT population, would represent/code only a few of such visual properties. Therefore, the goal of this analysis was not to find a few stimulus dimensions that could account for most of the variability in the representation of the visual objects. Rather, our goal was to check whether any principal component existed that could be associated to the variation of some global visual property across the object set. Interestingly, plotting the objects in the 2-dimensional space defined by the first two principal components revealed a trend that was consistent with the dendrogram obtained by hierarchical clustering. Namely, objects were distributed along the first principal component axis according to a gradient in object area, with large objects at one end of the axis and thin objects at the other end (see Fig. 3D). This trend was confirmed by showing that object area and object raking along the first principal component axis were highly and significantly anticorrelated (*r* = −0.69, *p*<0.001, *t*-test; see Fig. 3E). Similarly, object luminance (defined in the Materials and Methods) was significantly anticorrelated with the third principal component (*r* = −0.46, *p*<0.001, t-test). No significant correlation was found between the second principal component and any other low-level visual property considered in this study (i.e., contrast and aspect ratio, as defined in the Materials and Methods).

Overall, the analyses shown in Figure 3 indicate that visual objects, in the recorded IT neuronal representation space, were loosely segregated at the coarser (i.e., more “superordinate”) level according to a low-level visual property – object area (not to be confused with object size, which, in this study, was kept constant to 2° of visual angle for every object, and which is defined as the diameter of the larger circle fully enclosing the object).

### Definition of three alternative clustering hypotheses

To gain further insight into the principles underlying the grouping of visual objects in the recorded neuronal representation, we divided the object set in categories, according to three different clustering hypotheses: 1) shared semantic membership; 2) shared shape features (i.e., shape similarity); and 3) shared low-level visual properties.

Eleven semantic categories were built – four-limbed animals, birds, faces, fishes, insects, sea invertebrates, trees, vehicles, tools, music instruments and buildings (see Fig. S1A). The two superordinate semantic categories of animate and inanimate objects (which included, respectively, the first 6 and last 5 subordinate categories listed above) were also considered. All the semantic categories were built according to criteria established in previous studies [14], [15] (e.g., the trees were included in the inanimate category).

Fifteen categories of objects sharing visual shape features (named *shape-based categories* in the following) were defined as the 15 clusters obtained by running a *k*-means clustering algorithm over the objects' representation provided by the output layer of a brain-inspired object recognition model [43], [44] (see Materials and Methods for details). Each of these categories/clusters contained objects that occupied nearby positions (and were, therefore, similar) in the representational space of the object recognition model. Being such a similarity measured in a high-dimensional multivariate representation, it is impossible to precisely know what shared features brought two objects to cluster in the same category. Therefore, the shape-based categories were simply labeled by sequential numbers (from 1 to 15; see Fig. S1B). However, when the shape features underlying formation of a given category could be guessed by visual inspection, we assigned to such a category a descriptive name (e.g., the *round* objects' category or the *horizontal thin* objects' category). It should be kept in mind that these names are only used for the sake of readability, but they cannot possibly capture the true combinations of shape features underlying object clustering in the model representational space.

Eight Categories of objects sharing low-level visual properties (named *low-level categories* in the following) were defined on the base of four global properties of the images of the objects – luminance, contrast, area and aspect ratio (defined in the Materials and Methods). Each category contained 15 images having either the highest or the lowest values of one of such properties (see Fig. S1C).

It should be emphasized that no rigorous (or agreed-upon) definition exists of what should be considered low-level and high-level in terms of visual feature complexity. For this reason, our definitions of shape-based and low-level categories are essentially operational. That is, they refer to the complexity of the image processing that was performed to obtain them. In the case of the shape-based categories, the images of the objects were processed by banks of nonlinear filters in a multi-layered, feed-forward neural network (see Materials and Methods). Since these filters, collectively, extract visual features across a wide spectrum of complexity, the resulting shape-based categories included not only sets of moderately-to-highly complex visual patterns (such as round, oriented or star-like shapes), but also object sets that appeared to be defined mainly (but not exclusively) by lower-level image properties (such as contrast, luminance or texture). In the case of the low-level categories, the defining features were global image properties that could simply be extracted by segmenting the foreground image from the uniform-gray background. However, some of these properties, such as aspect ratio, can arguably be considered as moderately complex shape features. As a result, a few of the shaped-based categories substantially overlapped with the low-level categories and were assigned similar names (e.g., the *bright* and the *dim* shape-based categories partially overlapped, respectively, with the *high-luminance* and the *low-contrast* low-level categories; compare Figs. S1B and C). Such an overlap should not sound surprising, since the terms *shape-based* and *low-level* refer to the complexity of the operations underlying the definition of the categories, rather than to the content of the resulting categories. More in general, it should be stressed that the assessment of shape coding carried out in this study did not aim at precisely identifying what visual features were critical to elicit a response in specific neurons (or neuronal subpopulations). While methods to extract critical visual features exist (e.g., reverse correlation, image classification, or other fitting procedures of neuronal/behavioral responses to image properties [7], [8], [45]–[51]), the goal of our analysis was to assess how well various sets of visually similar objects clustered in the neuronal representation space, no matter whether visual similarity could be precisely defined in terms of specific visual properties (as in the case of the low-level categories) or not (as in the case of the shape-based categories).

### Overlap between clustering hypotheses and *k*-means clusters in the IT neuronal space

Having defined object categories based on three different hypotheses, we assessed to what extent the members of each category occupied nearby positions in the neuronal representation space. This was achieved by applying a *k*-means clustering algorithm to the neuronal population vectors, with the number of clusters *k* set to 15 according to both a Bayes and an Akaike Information Criterion [52] (hence, the choice of using such a number also in the *k*-means procedure that lead to the definition of the shape-based categories shown in Fig. S1B; see previous section). The resulting clusters were then compared to the semantic and visual similarity-based categories defined in the previous section, to check for any possible substantial overlap.

**Figure 4:**
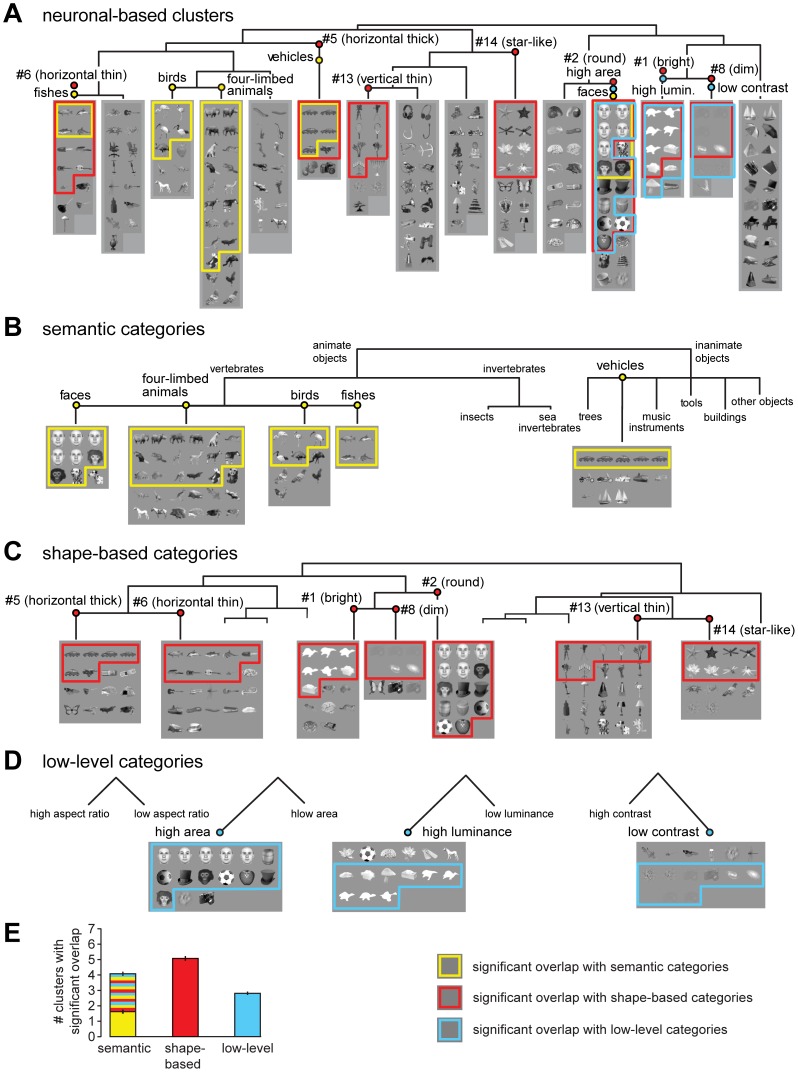
Overlap between *k*-means clusters in the IT neuronal space and object categories of the clustering hypotheses. (A) Fifteen object clusters obtained by a typical run of the *k*-means algorithm over the IT neuronal representation space. The clusters' arrangement was determined by applying a hierarchical clustering algorithm to their centroids (see the dendrogram on the top; the same approach was used to arrange the shape-based categories shown in C, which resulted from the *k*-means object clustering in the output layer of an object recognition model [44]). (B–D) The semantic (B), shape-based (C) and low-level (D) categories that significantly overlapped with some of the neuronal-based clusters shown in A. Overlapping neuronal-based clusters and categories are indicated by matching names (e.g., *faces*) in A and B–D, with the objects in common between a cluster and a category enclosed by either a yellow (semantic), a red (shape-based) or a cyan (low-level) frame. (E) Average number of significant overlaps between neuronal-based clusters and semantic (first bar), shape-based (second bar) and low-level (third bar) categories across 1,000 runs of the *k*-means algorithm over both the neuronal representation space and the model representation space. The yellow, red and cyan striped portion of the first bar indicates the number of neuronal-based clusters that significantly overlapped with both a semantic category and either a shape-based or a low-level category.

Figure 4A shows 15 object clusters that were obtained by a typical run of the *k*-means algorithm over the neuronal representation space (the *k*-means is not deterministic, therefore each run produces slightly different partitions of the data set; see below for further discussion). The order of the clusters in the figure was determined by applying an agglomerative hierarchical clustering algorithm to their centroids. This produced the dendrogram shown at the top of the figure, which allows appreciating the relationship among the *k*-means clusters (i.e., neighboring clusters in Fig. 4A lie nearby in the neural representation space). These clusters (named *neuronal-based clusters* in the following) were compared to the object categories of the three clustering hypotheses defined previously, some of which are shown in Figure 4B–D (all the categories are shown in Fig. S1). This was achieved by defining an overlap score that measured the fraction of objects in common between any given neuronal-based cluster and any given category in the three hypotheses. For easier comparison with Kiani et al., 2007, the same score defined in that study was used (see Materials and Methods; statistical significance of the overlap was computed through a permutation test, with Bonferroni corrected significance level *p*<0.05).

Figure 4 shows what neuronal-based clusters (A), on the one hand, and what semantic (B; yellow frames), shape-based (C; red frames) and low-level (D; cyan frames) categories, on the other hand, significantly overlapped (objects belonging to both a neuronal-based cluster and its matching category are shown within the corresponding frames; see the descriptive names on top of each cluster/category in A-D to navigate the figure and find matches between neuronal-based clusters and categories). Out of the fifteen neuronal-based clusters, five significantly overlapped with a semantic category, seven with a shape-based category, and three with a low-level category. Interestingly, some clusters significantly overlapped with multiple categories, each belonging to a different clustering hypothesis. For instance, the first cluster shown in Figure 4A overlapped both with the semantic category of *fishes* (forth category in Fig. 4B) and with the shape-based category #6 (that we named *horizontal thin*; see the second category in Fig. 4C). Similarly, the twelfth cluster in Figure 4A overlapped with both the semantic category of *faces* (first category in Fig. 4B), the shape-based category #2 (that we named *round*; see the fifth category in Fig. 4C), and the low-level category of *high area* objects (first category in Fig. 4D). Noticeably, in all these cases, the overlap was larger with the shape-based (or low-level) category than with the semantic category. Moreover, the objects overlapping with the semantic category were typically a subset of the objects overlapping with the shape-based category (see how the yellow frames are included within the red/cyan frames in Fig. 4A). That is, the objects belonging to a given semantic category were typically embedded within a larger group of objects with similar shape but different semantic membership (e.g., the fishes were embedded within a set of similarly horizontally elongated shapes, while the faces were embedded within a set of similarly round shapes). This implies that shape similarity (e.g., roundness) and not semantic membership (e.g., being a face) was at the root of these clusters within the neuronal representation space. On the other hand, a few neuronal-based clusters were found that significantly overlapped only with a semantic category. This is the case of the third and forth clusters in Figure 4A, which overlapped, respectively, with the *birds* (third category in Fig. 4B) and the *four-limbed animals* (second category in Fig. 4B).

To obtain a more robust assessment of what fraction of neuronal-based clusters significantly overlapped with categories of the three hypotheses and, in particular, how often semantic membership could be taken as the only explanation of the observed clusters, 1,000 runs of the *k*-means algorithm were performed (this produced 1,000 slightly different neuronal-based clusters and shape-based categories; the semantic and low-level categories were unchanged, since they were not obtained by a *k*-means procedure). Figure 4E shows the average number of neuronal-based clusters that, across these 1,000 *k*-means runs, significantly overlapped with categories of the three hypotheses. On average, about four, five and three clusters were found that significantly overlapped, respectively, with semantic, shape-based and low-level categories. Noticeably, more than half of the clusters that significantly overlapped with a semantic category, did so also with one of the categories defined by visual object similarity (see the yellow, red and cyan striped portion of the first bar in Fig. 4E). In all such cases, since the overlap was larger with the similarity-based category than with the semantic category, semantic membership cannot be taken as the factor at the root of object clustering in the neuronal representation. Rather, it is visual similarity among the members of those semantic categories that is driving object clustering.

Finally, to further test whether animate and inanimate objects were significantly segregated in the IT representation, 100 *k*-means runs were performed with *k* = 2, and the average absolute difference between the fraction of animate objects in the two clusters produced by each *k*-means run was computed. Such a difference amounted to ∼7% and was not significantly larger than expected by chance (i.e., by randomly shuffling the animate and inanimate objects among the clusters produced by each *k*-means run; *p* = 0.39), thus confirming the result of the analysis based on hierarchical clustering (see Fig. 3B).

Overall, the *k*-means analysis strongly suggests that most object clusters in the recorded IT neuronal representation are explainable by the visual similarity of their members at the level of both shape and, more surprisingly (being IT the highest purely visual brain area), low-level visual properties. Nevertheless, at least a couple of semantic categories exist (i.e., the *four-limbed animals* and the *birds*), whose significant representation in the recorded neuronal population is not accounted by either the shape-based or the low-level visual similarity metrics we used.

### Overlap between clustering hypotheses and D-MST clusters in the IT neuronal space

**Figure 5:**
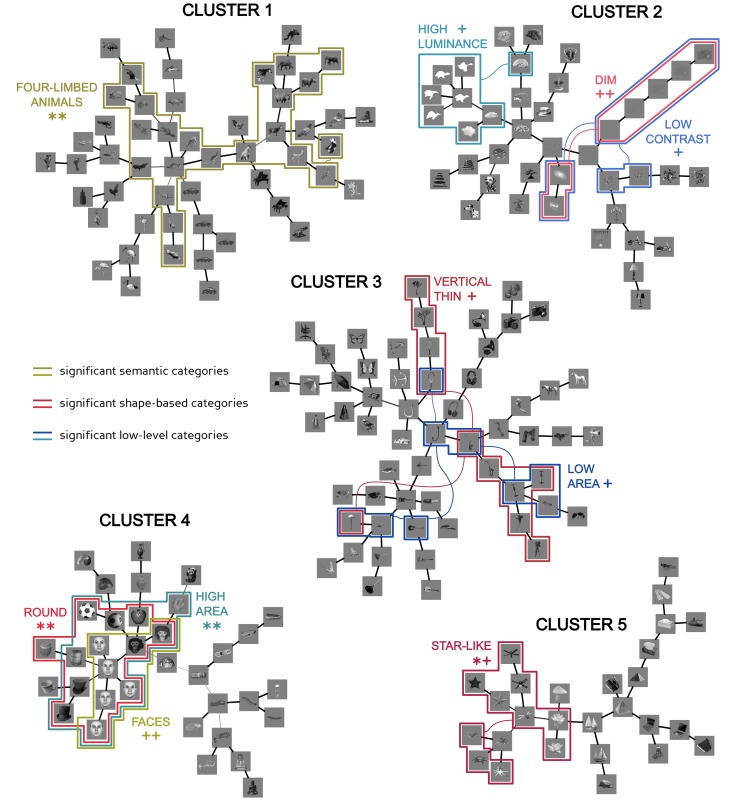
Overlap between D-MST clusters in the IT neuronal space and object categories of the clustering hypotheses. The five most stable clusters resulting from applying the D-MST clustering algorithm to the IT object representation (see also Fig. S2). The colored frames indicate the subsets of objects that, within each cluster, significantly overlapped with a semantic, a shape-based or a low-level category. The name of the overlapping category is reported near to each frame, together with the overlap's significance level (same overlap score and significance level symbols as in Table 1). The width and shade of the links connecting the images reflect the robustness of the links across different runs of the D-MST algorithm: thinner/lighter links appeared less frequently in the D-MST outcome with respect to thicker/darker links.

As a more refined way to infer the structure of visual object representation in IT, we sought an unsupervised approach that would embody the advantages of *k*-means-like partition algorithms (which allow measuring the fraction of overlapping objects between neuronal-based clusters and arbitrary object categories; see Fig. 4) and hierarchical approaches (which allow assessing the fine-grain relationship between objects within the representation space; see Fig. 3A). This was achieved by applying a method that has been recently developed in the domain of statistical physics – the D-MST clustering algorithm [53], [54]. This method interpolates between Affinity Propagation (a recent, state-of-the art partition algorithm that has been successfully applied in a variety of contexts [55]–[59]) and hierarchical Single Linkage clustering [60], [61]. The main advantage of the D-MST method over the *k*-means (and similar partition methods, such as Affinity Propagation) is to allow non-spherical clusters, i.e., to allow loosening the implicit assumption that all the elements of a cluster lie within some distance to some point (i.e., the centre of the cluster). In fact, the output of this method is not simply a partition of the elements into clusters, but, rather, it is a forest, i.e., a partition of the elements into trees (see Fig. 5). As a result, the outcome of the D-MST algorithm contains richer information about the topology/structure of the data, as compared to the output of the *k*-means (see Materials and Methods).

Figure 5 shows the five most stable clusters (see also Fig. S2 and Text S1) extracted by the D-MST algorithm from the recorded IT object representation (named *neuronal-based clusters* in the following). The fact that the number of D-MST clusters was much lower than the optimal number of *k*-means clusters (see previous section and Fig. 4A) is not surprising – the nature of these two clustering methods is very different, and the number of clusters they yield cannot be directly compared. In fact, the D-MST clusters have an inner hierarchical structure that incorporates as sub-trees what partition methods (such as the *k*-means) would segregate into separate clusters. The advantage of the D-MST approach is to make explicit the relationship among such sub-trees, thus providing additional topological information that, with other methods, would be lost. This can be appreciated by inspecting, for example, cluster #3, which is mostly made of objects with low area, but with different sub-trees containing objects with different features (e.g., vertically oriented edges, horizontally oriented edges, curved boundaries, etc.); or cluster #4, which is made of two distinct sub-trees, one containing round objects and another containing horizontally elongated objects; or cluster #5, in which there is a transition from star-shaped objects (on the left sub-trees) to objects containing sharp edges (on the right sub-trees), passing through a central region of spiky objects.

Figure 5 also shows what subsets of objects, within each cluster, significantly overlapped with one of the object categories of the clustering hypotheses. Critically, the significance of the overlap was computed through a permutation test that took into account the unrooted-tree internal structure of the D-MST clusters and the existence of *twin* objects (i.e., the fact that, as in [14], [15], our object set contained at least two exemplars/twins of any given object – two horses, two hats, two monkey faces, five human faces, etc; see Fig. 2). This was achieved by measuring the fraction of overlapping objects between a given category and all possible sub-trees of contiguous objects within a cluster, with the significance of the overlap assessed by randomly permuting sets of *twin* objects across the categories of a given clustering hypotheses (1,000,000 permutations were run; see Text S1 for details). Twins' sets, rather than individual objects, were permuted, because visual inspection of Figure 5 revealed that twins had a strong tendency to lie nearby in the IT representation space (i.e., a strong tendency to be directly connected in the D-MST clusters). This is not surprising, since twins are, in general, very similar at the pixel level, and, as a result, they typically belong to the same shape-based and low-level category, beside belonging, by definition, to the same semantic category (see Fig. S1). Therefore, the presence of twins tends to inflate the overlap between sub-trees within the D-MST clusters and object categories. Permuting twins' sets, rather than individual objects, allows taking into account this bias in the construction of the null distributions of overlap scores, against which the measured overlaps are compared to establish their significance. This yields a very conservative test, in which each set of twins counts as a single object, thus removing, de facto, any contribution of pixel-level similarity among twins to the computation of chance overlap scores.

As expected, this approach provided a very conservative outcome: only a few categories of the clustering hypotheses were found that significantly overlapped with sub-trees within the D-MST clusters (Holm-Bonferroni corrected ** *p*<0.01 and * *p*<0.05; see third-to-last column in Tables 1–3). This number increased if the Holm-Bonferroni correction was released, yielding two, four and four significant overlaps with categories, respectively, of the semantic, the shape-based and the low-level hypotheses (see third-to-last column in Tables 1–3 and corresponding yellowish, reddish and bluish frames in Fig. 5).

**Table 1:** Overlapping between semantic categories and D-MST neuronal-based clusters.

Category	D-MST Cluster	Ratio 1	Ratio 2	Overlap	*p* (twins)	Signif.	p (obj.)	Signif.
Four-limb. anim.	1	0.73	0.96	0.71	0.0000	**	0.0000	**
Faces	4	0.78	1.00	0.78	0.0023	++	0.0000	**
Fishes	1	0.75	1.00	0.75	0.0742		0.0007	*+
Sea invertebr.	5	0.50	0.86	0.46	0.0840		0.0004	**
Birds	1	1.00	0.48	0.48	0.1048		0.0003	**
Music instr.	3	0.50	0.75	0.43	0.1140		0.0012	*+
Vehicles	1	0.46	0.67	0.37	0.2617		0.0065	++
Insects	3	0.58	0.47	0.35	0.3635		0.0192	+
Tools	3	0.58	0.44	0.33	0.4587		0.0365	+
Trees	5	0.30	1.00	0.30	0.6240		0.0979	
Buildings	5	0.33	1.00	0.33	0.8883		0.1471	

The table reports the overlap (fifth column) between each semantic category (first column) and the D-MST neuronal-based cluster (second column) containing the best matching sub-tree of contiguous objects, according to a score defined as the ratio between the intersection of the sub-tree with the category and their union (fifth column). Significance of the overlap was computed by permuting (1,000,000 times) either sets of twin objects (forth- and third-to-last columns) or individual objects (second-to-last and last columns) across the categories of a given clustering hypotheses: Holm-Bonferroni corrected *p*<0.01 (**) and *p*<0.05 (* and *+); and uncorrected *p*<0.01 (++ and *+) and *p*<0.05 (+). For comparison with [14], two other overlap metrics (Ratio 1 = the fraction of objects in the category overlapping with the cluster; and Ratio 2 = the fraction of objects in the cluster overlapping with the category) are also reported.

**Table 2:** Overlapping between shape-based categories and D-MST neuronal-based clusters.

Category	D-MST Cluster	Ratio 1	Ratio 2	Overlap	*p* (twins)	Signif.	*p* (obj.)	Signif.
#2 (round)	4	1.00	1.00	1.00	0.0000	**	0.0000	**
#14 (star-like)	5	0.71	0.91	0.67	0.0007	*+	0.0000	**
#8 (dim)	2	0.78	0.78	0.64	0.0097	++	0.0000	**
#13 (vertical thin)	3	0.52	0.68	0.42	0.0347	+	0.0002	**
#6 (horiz. thin)	3	0.41	1.00	0.41	0.0520		0.0003	**
#1 (bright)	2	0.57	0.66	0.44	0.0748		0.0004	**
#5 (horiz. thick)	1	0.44	0.87	0.41	0.0927		0.0008	*+
#12 (diagonal)	1	0.47	0.50	0.32	0.4299		0.0392	+
#15	1	0.50	0.50	0.33	0.4878		0.0368	+
#10	3	0.45	0.50	0.31	0.5313		0.0667	
#11	3	0.31	1.00	0.30	0.5347		0.0582	
#4	1	0.45	0.41	0.28	0.7109		0.1694	
#7 (pointy)	5	0.27	0.60	0.23	0.9279		0.4949	
#9	1	0.29	0.50	0.22	0.9451		0.5630	
#3	2	0.33	0.40	0.22	0.9530		0.5768	

The table reports the overlap (fifth column) between each shape-based category (first column) and the D-MST neuronal-based cluster (second column) containing the best matching sub-tree of contiguous objects. Same table structure and symbols as in Table 1.

**Table 3:** Overlapping between low-level categories and D-MST neuronal-based clusters.

Category	D-MST Cluster	Ratio 1	Ratio 2	Overlap	*p* (twins)	Signif.	*p* (obj.)	Signif.
High area	4	0.93	1.00	0.93	0.0000	**	0.0000	**
Low contrast	2	0.60	0.82	0.53	0.0103	+	0.0000	**
Low area	3	0.60	0.69	0.47	0.0333	+	0.0001	**
High luminance	2	0.53	0.80	0.47	0.0352	+	0.0001	**
Low aspect ratio	2	0.40	0.86	0.37	0.1910		0.0049	++
High aspect ratio	4	0.33	0.83	0.31	0.4760		0.0454	+
Low luminance	1	0.33	0.42	0.28	0.9240		0.5116	
High contrast	1	0.33	0.36	0.21	0.9761		0.7167	

The table reports the overlap (fifth column) between each low-level category (first column) and the D-MST neuronal-based cluster (second column) containing the best matching sub-tree of contiguous objects. Same table structure and symbols as in Table 1.

Noticeably, out of the two semantic categories that significantly overlapped with a sub-tree within a D-MST cluster, only for the *four-limbed animals* (in cluster #1) such an overlap was not accountable by the similarity of their members, since the *faces* (in cluster #4) were part of a larger sub-tree of *round* objects with *high area*. Moreover, although cluster #1 contained both a large subset of *four-limbed animals* and a large subset of *birds*, only the former was compactly represented, while the latter was very scattered, thus suggesting that the proximity of the birds was mostly mediated by other objects in the cluster. Since our overlap measure took into account the compactness of a given object category within a tree (see above), no significant overlap between the *birds* category and any sub-tree within cluster #1 was found.

The results shown in Figure 5 provide a very robust and conservative assessment of what semantic and visual similarity-based categories were represented in our recorded IT population. However, in previous studies [14], the significance of the overlap between neuronal-based clusters and object categories was computed without compensating for the existence of multiple (very similar) exemplars of the same objects (i.e., twins). For easier comparison with such studies, we also computed the significance of the overlap scores reported in Tables 1–3 by randomly shuffling individual objects, rather than twins' sets. This yielded an additional set of semantic categories that significantly overlapped with sub-trees within the D-MST clusters – *birds*, *sea invertebrates*, *fishes* and *music instruments* (Holm-Bonferroni corrected ** *p*<0.01 and * *p*<0.05; see last column in Table 1). However, an even larger increase of overlaps was found between sub-trees within the D-MST clusters and shape-based and low-level object categories (see last column in Tables 2–3). Critically, in most cases, these overlaps with the visual similarity-based categories accounted for the overlaps with the semantic categories (the same way roundness accounted for the clustering of faces in cluster #4 of Fig. 5). In fact, the *sea invertebrates* were part of the larger cluster of *star-like* shapes in cluster #5; the *fishes* were part of the larger cluster of *horizontal thick* objects in cluster #1; and the *music instruments* were part of the larger cluster of *horizontal thin* objects in cluster #3 (cross-compare Fig. 5 and the third-to-last and last columns of Tables 1–2). Therefore, regardless of the level of conservativeness of the permutation test, the D-MST clustering analysis strongly suggests that visual similarity, rather than semantic membership, was at the root of the structure of visual object representations in the recorded IT population (with the noticeable exception to the *four-limbed animals* and, to a lesser extent, the *birds* semantic categories).

This conclusion was strengthened by the qualitative observation of the D-MST clusters, whose internal structure provided a richness of information that was not always captured by our overlap and similarity metrics. For instance, four-legged grand-pianos and four-wheeled cars (among other inanimate objects) belonged to the same cluster of the four-limbed animals, thus suggesting that some shared, hard-to-quantify visual property, rather than semantic membership, may have underlain the grouping of objects in cluster #1. Similarly, shared visual features likely played a relevant role in determining the clustering of other groups of objects (see, for instance, the objects with high spatial frequency texture/patterns in tree #2, or the objects with curved or round elements in tree #3).

Overall, the object clustering produced by the D-MST algorithm suggests the existence of a rich multi-level object representation in IT, which is largely driven by the similarity of visual objects across a spectrum of visual properties, ranging from low-level image attributes to complex combinations of shape features that are often hard to model and quantify.

### Read-out of object category membership from the IT population activity

Unsupervised approaches, such as the clustering methods described in the previous sections, have the main advantage of discovering the “natural” internal structure of neuronal object representations, but do not provide a direct assessment of how much information a neuronal population conveys about a given object set (e.g., a semantic or a visual similarity-based category). In addition, since they are based on average firing rates computed in a time epoch following stimulus presentation, they do not take into account the trial-by-trial variability of neuronal responses [62], [63]. As an alternative, a useful tool to directly estimate the representational power of a neuronal population (and take into account trial-by-trial response variability) is provided by supervised decoding approaches, such as discriminant-based linear classifiers [62], [64]–[67]. These approaches are particularly appealing when dealing with neuronal representations, since they are based on linear read-out schemes that are plausibly implementable by the neuronal machinery.

We estimated the power of the recorded IT population to support classification of the objects belonging to the categories of the clustering hypotheses, by building binary Fisher Linear Discriminants (FLDs) [60]. The FLDs were trained to learn the mapping between the neuronal population response vectors and the labels that were assigned to each object according to a given binary classification task (e.g., faces vs. all other objects in the set). We then measured the performance of the classifiers at generalizing to novel population responses (i.e., at correctly labeling left-out population vectors that were not used during training), using standard cross-validation procedures to establish the variability and significance of the classification performance (see Materials and Methods). Specifically, we tested the capability of the FLDs to correctly classify visual objects that were not used to build (i.e., train) the classifiers. That is, all the population vectors obtained across different presentations of a given object in a given category (e.g., a given face in the *faces* category) were excluded from the training set, and one of such left-out population vectors was used to test the classifier performance in the cross-validation procedure.

**Figure 6:**
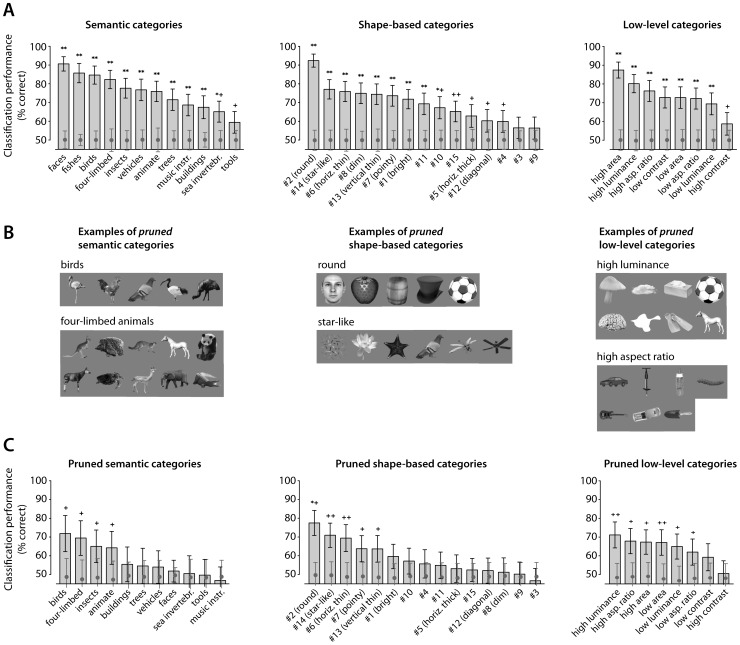
Fisher Linear Discriminant (FLD) analysis of IT population activity. (A) Each gray bar reports the average performance of a binary FLD at correctly classifying members of a given object category (e.g., faces) from all other objects in the set. For each binary classification task, the standard deviation of the performance (error bars), and the mean and standard deviation of the null distribution (gray circles and their error bars), against which significant deviation of performance from chance was assessed (same significance level symbols as in Table 1), are also reported (see Materials and Methods for a description of the cross-validation and permutation procedures yielding these summary statistics). (B) Examples of “pruned” semantic, shape-based and low-level categories that were obtained by subsampling the original object categories (shown in Fig. S1), so as to minimize the overlap between semantic and visual information (see Materials and Methods for details). (C) Performance of the FLDs at correctly classifying members of the pruned categories (same symbols as in A).

The average classification performance of the FLDs was significantly higher than what expected by randomly permuting the object labels (*p*<0.05; see Materials and Methods for details) for all the semantic categories, most of the shape-based categories (13 out of 15), and all the low-level categories (see Fig. 6A). At first, this result may seem surprising (and at odd with our previous analyses; see Figs. 3–5), but it can be easily understood, by considering the existence of multiple (very similar) exemplars of the same objects (i.e., the twins) in our stimulus set (see Fig. 2). Indeed, the large (and significant) classification performance obtained for virtually all the FLDs in Figure 6A is fully consistent with the large number of significant overlaps between D-MST clusters and object categories reported in the last column of Tables 1–3 (i.e., when the significance of the overlap was computed without compensating for the existence of twins).

To understand how twins can explain the high performances of the FLDs, it should be recalled that, as shown by the D-MST clusters (see Fig. 5), twins typically lay nearby in the neuronal representation space. Therefore, it is not surprising that an FLD, trained to classify a given member in pair of twins, successfully classifies the other member of the pair (when this member is used as the left-out test object). The problem is that, for most twins, it is impossible to know whether it is their shared semantic membership or their visual similarity that drives their clustering in the neuronal space (and, therefore, the high performance of the FLDs). In fact, twins belong, by construction, to the same semantic category (see Fig. S1A), but, in most cases, they also belong to the same shape-based or low-level category (see Figs. S1B–C), being twins, in general, very similar, in terms of shape, orientation, pose, contrast, luminance, etc (compare adjacent objects in Fig. 2).

The issue with twins brings up the more general issue of how to fully disentangle the contributions of semantic membership and shape similarity to the establishment of cortical visual object representations, when sets of natural objects (containing many similar members of the same semantic categories) are used to probe such representations. To tackle this issue, and better dissociate semantic information from visual information, we subsampled/pruned the object categories, so as to obtain semantic categories made only of dissimilar objects, and shape-based/low-level categories made only of objects with different semantic membership. This was achieved by imposing the constraints that: 1) no pair of objects in any given semantic category belonged to the same shape-based or low-level category; 2) no pair of objects in any given shape-based or low-level category belonged to the same semantic category; and 3) only a single exemplar of any set of twins (e.g., a single human face or a single hat) belonged to any given category. Since many different “pruned” categories could be obtained from any of the original object categories, the subsampling procedure was repeated many times (once for each cross-validation run; see Materials and Methods for details; examples of pruned categories are shown Fig. 6B). We then measured the performance of the FLDs at correctly classifying left-out objects from such pruned categories (see Fig. 6C).

As expected, the classification performance of the FLDs was much reduced, as compared to what obtained with the original categories (compare Figs. 6A and C). Only three of the subordinate semantic categories (*birds*, *four-limbed animals*, and *insects*; see Fig. 6C, first panel) were classified with a performance that was higher than what expected by chance (*p*<0.05, permutation test; see Materials and Methods for details). In addition, the animate category (as a whole) was discriminated with higher than chance performance from the inanimate category. Among the categories defined by visual similarity, five shape-based categories (*round*, *star-like*, *horizontal thin, pointy* and *vertical thin* objects), as well as six low-level categories (*high* and *low area*, *high* and *low luminance* and *high* and *low aspect ratio* objects), were all classified with higher than chance performance by the FLDs (see second and third panels in Figs. 6C). Among all tested categories, the highest classification performance (>75% correct) was obtained for the shape-based category of *round* objects (this was the only performance to remain significantly higher than chance, after that a Bonferroni correction for multiple comparisons was applied).

Overall, the result of the FLD analysis, applied to the pruned categories, was in good agreement with the result of the D-MST clustering, when significance was computed by permuting twins' sets (see Fig. 5 and Tables 1–3, third-to-last column). Comparing the outcome of the two analyses (see Tables 4–6), only a few differences emerged. For instance, the *insects* (among the semantic categories) and the *pointy objects* (among the shape-based categories) were significantly represented in the neuronal space according to the FLD analysis, but not according to the D-MST. Similarly, the animate and inanimate categories were linearly separable according to the FLD analysis, although animate and inanimate objects were not sharply segregated in different D-MST clusters (as also shown by the hierarchical clustering and *k*-means analysis; see Figs. 3A-B and 4). Such discrepancies are not surprising, since, in general, supervised and unsupervised multivariate approaches provide complementary information about data representations – for instance, linear separability (as measured by FLDs' classification performance) is not bound to perfectly match the clustering of data in a representational space (see further comments in the Discussion). Hence, the importance of combing both kinds of approaches when exploring a multivariate data set. When this was done, and the outcomes of the D-MST and FLD analyses were taken together, a very conservative assessment of what object categories were represented by the recorded IT population was achieved (see last column in Tables 4–6) – one semantic category (the *four-limbed animals*), three shape-based categories (*round*, *star-like* and *vertical thin* objects), and three low-level categories (*high area*, *low area* and *high luminance*) turned out to be significantly represented according to both approaches. Overall, this confirmed that visual similarity (at the level of both shape and lower-order properties) accounted for the neuronal representation of visual objects better than semantic membership did.

**Table 4:** Semantic categories significantly represented in IT according to the D-MST and the FLD analyses.

Category	Signif. D-MST (twins' sets perm.)	Signif. FLD (pruned cat.)	Signif. D-MST & FLD
Four-limb. anim.	**	+	✓
Faces	++		
Birds		+	
Insects		+	

The second and third columns report what semantic categories were found to be significantly represented in IT according, respectively, to the D-MST analysis (when significance was computed by permuting twins' sets; i.e., same data as in Fig. 5 and in the third-to-last column of Table 1) and to the FLD analysis (when classifiers were applied to the pruned object categories; i.e., same data as in Fig. 6C). Same significance level symbols as in Table 1. The last column shows what semantic categories were found to be significantly represented in IT according to both the D-MST and the FLD analyses.

**Table 5:** Shape-based categories significantly represented in IT according to the D-MST and the FLD analyses.

Category	Signif. D-MST (twins' sets perm.)	Signif. FLD (pruned cat.)	Signif. D-MST & FLD
#2 (round)	**	*+	✓
#14 (star-like)	*+	++	✓
#8 (dim)	++		
#13 (vertical thin)	+	+	✓
#6 (horiz. thin)		++	
#7 (pointy)		+	

The second and third columns report what shape-based categories were found to be significantly represented in IT according, respectively, to the D-MST analysis (when significance was computed by permuting twins' sets; i.e., same data as in Fig. 5 and in the third-to-last column of Table 2) and to the FLD analysis (when classifiers were applied to the pruned object categories; i.e., same data as in Fig. 6C). Same significance level symbols as in Table 1. The last column shows what shape-based categories were found to be significantly represented in IT according to both the D-MST and the FLD analyses.

**Table 6:** Low-level categories significantly represented in IT according to the D-MST and the FLD analyses.

Category	Signif. D-MST (twins' sets perm.)	Signif. FLD (pruned cat.)	Signif. D-MST & FLD
High area	**	+	✓
Low contrast	+		
Low area	+	++	✓
High luminance	+	++	✓
Low aspect ratio		+	
High aspect ratio		+	
Low luminance		+	

The second and third columns report what low-level categories were found to be significantly represented in IT according, respectively, to the D-MST analysis (when significance was computed by permuting twins' sets; i.e., same data as in Fig. 5 and in the third-to-last column of Table 3) and to the FLD analysis (when classifiers were applied to the pruned object categories; i.e., same data as in Fig. 6C). Same significance level symbols as in Table 1. The last column shows what low-level categories were found to be significantly represented in IT according to both the D-MST and the FLD analyses.

## Discussion

This study investigated what visual object properties were represented in a neuronal population that was recorded from monkey inferotemporal cortex. To this aim, we defined three alternative hypotheses that could underlie the clustering of a battery of visual objects within the IT neuronal representation space: 1) shared semantic membership; 2) shared visual shape features (i.e., shape similarity); and 3) shared low-level visual properties. We then applied an array of unsupervised and supervised machine learning approaches to understand whether the object categories defined by these hypotheses were robustly represented in the recorded IT neuronal population. Based on these approaches, we concluded that the coarse clustering of visual objects in the neuronal representation space was mainly driven by low-level visual properties, while its finer-grain structure depended on higher-level shape features, with little role played by semantic membership (although our analyses cannot exclude that at least one semantic category – the *four-limbed animals* – was also robustly represented in the recorded IT population).

These conclusions are mostly in disagreement with those of two recent studies [14], [15] that also investigated the nature of object representations in monkey IT (and its human homologous). In these studies, the authors found a sharp segregation between animate and inanimate objects, and a finer-grain clustering within the animate category that matched closely several subordinates semantic categories (named “intuitive” or “human-conventional” categories by the authors), such as faces, body parts, four-limbed animals, fishes, reptiles, butterflies, etc. Most remarkably, these studies were unable to find any visual-similarity metric that could produce object clusters matching those found in the neuronal representation.

The conclusions reached by our study are consistent with [14], only as far as the representation of a few animate categories is concerned: the *four-limbed animals* (see Figs. 4, 5 and 6C, and Tables 1 and 4) and, to a lesser extent, the *birds* (see Figs. 4 and 6C, and Table 4). However, we did not find any other semantic category that was significantly represented in the recorded IT population according to all (as in the case of *four-limbed animals*) or most (as in the case of *birds*) the multivariate approaches we applied. For instance, the *insects* were found to be linearly discriminable by the FLDs (see Fig. 6C), but no compact clusters of insects were found by the *k*-means and the D-MST clustering algorithms. In the case of *faces*, their clustering in the neuronal representation space was accounted for by their visual similarity, rather than their shared semantic membership (as shown by the fact that faces were part of a larger cluster of objects with round shape and large area; see Figs. 4 and 5) – when pruned face categories made only of dissimilar faces were built, the FLDs were no longer able to correctly classify them (compare Fig. 6A and C). Finally, no sharp segregation between animate and inanimate objects was observed (but see further discussion below). On the other hand, we found several shape features and lower-level visual properties that successfully accounted for the clustering of some visual objects in the IT neuronal representation. Among others, the more prominent are: 1) object area, which determined the gross topology of object clustering in the IT representation (see Figs. 3C–E); 2) other low-level image properties, such as object luminance and aspect ratio (see Figs. 4–6 and Tables 3 and 6); 3) shape features, such as specific arrangements of edges and boundaries that defined round, horizontally elongated, vertically elongated and star-like objects (see Figs. 4–6 and Tables 2 and 5).

### Animate and inanimate objects are not sharply segregated in the IT representation

In our study, animate and inanimate objects were found to be equally distributed among the first two nodes of the dendrogram produced by hierarchical clustering (see Figs. 3A, B) and in the two clusters obtained by running the *k*-means algorithm with *k* = 2. In addition, most of the clusters produced by the *k*-means (Fig. 4) and D-MST (Fig. 5) algorithms contained a mixture of animate and inanimate objects. However, the FLDs were able to distinguish animate from inanimate objects with higher than chance performance, even after that visual similarity among members within each category was minimized (see Fig. 6C). The latter finding is not contradictory with the results of the cluster analyses, since it is indicative of the compactness of some subordinate semantic categories (such as the *four-limbed animals* and the *faces*; see Figs. 4 and 5), rather than of the superordinate animate category as a whole. In particular, FLDs, being supervised approaches, do not need to follow the “natural” object segregation in the IT representation (as revealed by the unsupervised clustering methods). Rather, given the high dimensionality of the representation space, FLDs could find a hyperplane segregating the two main animate groups (i.e., *four-limbed animals* and the *faces*) from the inanimate objects, even if those groups belong to different “natural” clusters.

In conclusion, our analysis strongly suggests that animate and inanimate objects are not sharply segregated within the IT representation, at least as we have sampled it here. At the same time, however, they are not randomly scattered across the IT neuronal space. Instead, some subordinate animate categories form compact clusters in the IT representation (although, in some cases, simply because of the visual similarity of their members). This conclusion, while being at odd with [14], [15], is in agreement with a recent fMRI study showing that, in the body-selective regions of monkey inferotemporal cortex, objects do not primarily segregate according to whether they belong to the animate or the inanimate categories [34].

### Comparison with other studies

The discrepancy between our and previous results [14], [15] is not easily explained. The stimulus presentation protocols (monkeys viewing images presented in rapid sequence) and the region from which the neuronal responses were recorded (anterior IT) are comparable (although not fully overlapping; see further discussion below). The analytical approaches are at least partially overlapping, although in our study more advanced tools derived from statistical mechanics were used.

One potentially important difference is the way in which statistical significance of the overlap between the object categories and the neuronal-based clusters was evaluated. We took into account the effect of having sets of very similar exemplars of the same objects (i.e., twin objects) on the outcome of the statistical tests (see Fig. 5 and the third-to-last row in Tables 1–3). We also tried to fully dissociate the representation of visual similarity and semantic membership by building semantic categories that contained only very dissimilar objects, and shape-based categories that contained only objects with different semantic membership (see Fig. 6B–C). As far as we understand, the effect of twins on the overlap score was not taken into account by Kiani and colleagues [14], in spite of the many different exemplars of the same objects contained in their object set. As shown by our results, shuffling objects rather than twins' sets in the statistical analysis, dramatically increased the number of significant overlaps between D-MST neuronal-based clusters and object categories (compare the third-to-last and last columns in Tables 1–3). The impact of shape similarity on the representation of semantic categories was shown to be even more dramatic in the case of the FLD analysis – minimizing shape similarity within semantic categories dramatically reduced the number of categories, whose elements were classified with higher than chance performance by FLDs (compare the first panels in Fig. 6A and Fig. 6C).

The failure of the visual similarity metrics used by [14], [15] to account for object clustering in IT could be explained by the different metrics used in their studies and ours. In particular, although we used the same object recognition model [43], [68] to quantify shape similarity, our implementation of the model included a much larger number of output units (24,451) as compared to [14] (674). In fact, we did not try to match the number of model output units to the number of recorded neurons (as done by [14]), since our goal was not to model IT, but, rather, to find a metric that was as powerful as possible in capturing the visual shape similarity among the objects in our set.

Another substantial difference is represented by the stimulus set. The objects used in our experiments were grayscale pictures of natural objects, while, in the studies of [14], [15], color pictures were used. Color is obviously a very salient object feature that could have strongly influenced the object clustering reported in those studies. For instance, human faces, hands, body parts and, to a lesser extent, monkey faces, as well as the fur of many animals, all have a pink/brownish hue that could have driven their clustering in the superordinate category of animate objects. Noticeably, in the above-mentioned fMRI study that found no segregation between animate and inanimate objects, grayscale pictures were used [34]. In conclusion, the use of colorful images in [14], [15] represents a major confounding factor, since IT color tuning may interact with IT shape tuning in ways that are hard to quantify/model.

Yet another difference is the lower number of visual objects we tested (213), the smaller population of IT neurons we recorded (94), and the smaller extent of IT cortex we sampled, as compared to Kiani and colleagues (who tested 1,084 objects and recorded the responses of 674 IT neurons). These are three separate, but related, issues, each deserving a specific discussion.

While, in general, recording from a wider IT neuronal sample would lead to a more refined assessment of IT neuronal population coding, it is unclear whether major qualitative differences in the structure of visual object representations would emerge as a function of the size of the recorded neuronal pool. Previous investigations of population coding in IT have shown a gradual increase of the amount of information conveyed by a pool of IT neurons about object identity or category as a function of the pool size, but they have not reported any dramatic qualitative shift in what the neuronal pool would code depending on its size [30], [64], [66]. In addition, these studies have revealed that small pools of IT neurons can be as effective (or more effective) than much larger populations, as long as their selectivity for object identity or category is very strong. In this regard, it should be noted that Kiani and colleagues recorded every neuron they could isolate regardless of its stimulus responsiveness or selectivity, which could potentially have resulted in a neuronal pool with many unresponsive or non-selective cells (they report that 38% of their neurons were category selective). By contrast, we recorded only cells with a statistically reliable response to at least one of the objects in our stimulus set, thus obtaining a population of neurons with robust tuning across the tested objects (see [38]). Based on the above-mentioned population coding studies, this suggests that Kiani and colleagues' larger IT sample could only be marginally better than our smaller (but more selective) neuronal pool at estimating IT neuronal representations of visual objects (the large performances achieved by the FLDs in Fig. 6 confirm the effectiveness of the sampled IT population at conveying information about features/properties of our object set).

As far as the size of the stimulus set is concerned, it should be noted that a larger stimulus set does not necessarily mean a better stimulus set, when it comes to disentangling alternative clustering hypotheses. First, as pointed out above, a large number of very similar exemplars per category could lead to an overestimation of the significance of the overlap between neuronal-based clusters and, for instance, semantic categories, if not properly taken into account in the statistical analysis. Second, although our semantic categories typically contained less exemplars than those used by Kiani and colleagues, the superordinate categories of animate and animate objects used in our study contained a large number of exemplars. Nevertheless, as pointed out above, we did not found any sharp segregation of these two categories in the IT representation.

Finally, one factor that could explain some of the discrepancies between our conclusions and those of Kiani and colleagues is the different extent of IT cortex that was sampled in the two studies. Our recordings targeted the most medial part of the ventral bank of STS and of the ventral surface lateral to AMTS (see blue dots and red-shaded areas in Fig. 1) and spanned a 13–17 mm anteroposterior range, while Kiani and colleagues sampled a larger portion of IT, both mediolaterally (i.e., including the gyrus between STS and AMTS), and anteroposteriorly (i.e., a 13/15–20 mm span; see Fig. 1 in [14]). This suggests that recordings in [14] may have sampled sub-regions in IT that are known to contain enriched populations of face-selective cells (i.e., the anterior face patches AL and AM [33]; see Fig. 1), while, in our study, only a minimal overlap between recording sites and face patch AM could, in principle, be expected (in practice, our IT sample did not contain any cell that was sharply tuned for faces; see Fig. S3 and further discussion in the next Section). This could explain why in [14], differently from our study, a sharp clustering of human, monkey, and animal faces was found in the IT representation.

To conclude, it is hard to infer what methodological differences may be at the root of the discrepancies between our study and [14], [15]. Above, we have listed some of the differences that could be crucial. Ultimately, however, only a re-analysis of Kiani and colleagues' data with our analytical/statistical approaches, or, better, a full new set of recordings (e.g., with grayscale versions of the images used by Kiani and colleagues) could shed more light on the causes of these discrepancies. Both approaches are clearly beyond the scope of this study, but could be an interesting target of future investigations by ours or other groups.

### Validity and implications of our findings

As pointed out in a recent review [13], two main competing ideas exist about what kind of object information is coded by the ventral stream, and, in particular, by its highest stage – anterior IT. On the one hand, many single-unit studies in monkeys support the notion of structural (or shape-based) representations along the ventral stream – i.e., combinations of object-defining visual features of increasing complexity are coded along the ventral steam, with the highest complexity of configural coding reached in anterior IT (see [2], [4], [6] for a review). On the other hand, another line of evidence (mainly coming from human lesion and fMRI studies) supports the existence of semantic categorical representations along the ventral stream – i.e., human high-level representations of visual objects segregate according to object function/meaning rather than shape [17]–[24]. The findings reported by [14], [15] have added evidence based on monkey single-unit recordings to support the latter notion.

Our study, on the contrary, strongly supports the notion that inferotemporal neuronal ensembles, in the monkey brain, mainly represent visual, rather than semantic, information. In particular, our analyses show that IT response patterns code not only structural/configural shape information of various complexity, but also a whole array of low-level image properties (such a overall luminance, area, aspect ratio, etc.). On the one hand, this is surprising, since this kind of low-level information contained in the visual input is typically though to be extracted by lower-level visual areas and not to be preserved and coded in IT. On the other hand, previous computational and empirical studies have shown that object identity is represented along with other low-level properties in IT, namely position and size. In particular, it has been shown that IT neuronal ensembles can code not only object identity regardless of position/size 64–66 (thus conveying a position/size invariant object representation), but they can also code object position/size regardless of object identity [64], and can jointly code object position and identity [65] (i.e., report the identity of a specific object at a specific visual field location). Our findings not only confirm these previous conclusions (extending them to a larger set of low-level properties), but also show how, topologically, some low-level properties (e.g., area of the visual field subtended by each visual object) and higher-order shape features are co-represented in IT (with the former determining the gross topology of object representations and the latter determining their finer-grain structure).

As far as semantic categories are concerned, only for the four-limbed animals we observed a significant and robust representation of semantic membership that could not be accounted by their visual similarity (see Table 4). This could either be the result of an extremely transformation-invariant population code of animal-like objects (and, therefore, still shape-based, although not captured by our visual similarity metrics), or could reflect learned associations between objects with dissimilar shape but similar meaning/function. The latter hypothesis would be consistent with the finding that neurons in higher-order areas of both the ventral and the dorsal streams can learn to encode general categorical associations between arbitrary visual patterns [25]–[27], [69]–[71], and would support the notion that semantic (or categorical) representations do exist in monkey IT [30], at least for a few selected, behaviorally relevant categories.

This raises the issue of what object categories, in our stimulus set, can be considered as behaviorally relevant (or meaningful) for the monkeys. This is obviously an important issue, when considering the generality of our conclusions, since the failure to observe a significant representation for most semantic categories could be due to their lack of “meaning” for the monkeys. While some animate categories (such as the four-limbed animals and the faces) are likely meaningful for the monkeys (either because innately such, or because meaning may have been acquired through repeated exposure to members of these categories, e.g., other monkeys and humans), other categories (especially among the inanimate set) are likely arbitrary collections of objects for the monkeys. Regardless of their likely meaningfulness for the monkeys, there are three reasons why it was important for us to ask how well all these categories were represented in IT. First, our semantic categories were defined so as to match as close as possible those defined by Kiani and colleagues [14], who found a significant representation in IT not only for four-limbed animals and faces, but also for most other animate categories (i.e., birds, reptiles, butterflies, fishes, etc.). Moreover, although in [14] only one of the inanimate subordinate categories (cars) was found to be significantly represented in IT, the inanimate category, as a whole, was sharply segregated from the animate category. Since one of the goals of our study was to provide a comparison with the findings of Kiani and colleagues, it was essential to test how well the animate and inanimate categories, as well as all their possible subordinate categories were represented in IT. Second, our monkeys had a daily, prolonged exposure not only to other monkeys and humans, but also to a variety of inanimate objects, such as toys, fruits, vegetables, furniture, tools and equipment used in the animal facility and in the lab (some of which are similar to the inanimate objects contained of our stimulus set). Therefore, if the representation of visual objects in monkey IT is organized, at its most superordinate level, according to an animate/inanimate distinction (as concluded in [14], [15]), there is no reason to believe that the development of such an animate/inanimate segregation was precluded to our monkeys. Hence, the relevance of testing the existence of such a segregation and provide a comparison with [14]. Third, testing the representation of inanimate (but also animate) categories without any obvious meaning for the monkeys (e.g., music instruments or sea invertebrates; see Fig. S1) served as a demonstration that shape similarity among members of the same semantic category, if not properly taken into account in the statistical analysis, can easily lead to an overestimation of how well semantic membership is represented in visual cortex. This is shown by the many semantic categories that were found to have a significant representation in IT according to the D-MST and FLD analyses, unless shape similarity (e.g., the presence of twins) was properly accounted for (compare the significance levels in the third-to-last and last columns of Table 1, and compare Figs. 6A and C). In summary, testing the many animate and inanimate categories used in our study provides a valuable comparison with previous reports [14], [15] (e.g., about the animate/inanimate segregation), and cautions against giving semantic interpretations of cortical activity patterns that may actually reflect visual shape similarity. Finally, it is worth pointing out that, as a way to better understand to what extent behaviorally relevant categories are represented in monkey IT, future studies should first try to establish what objects are naturally perceived/judged by monkeys as belonging to the same categories (e.g., by relying on priming or adaptation aftereffect paradigms that allow measuring what objects are spontaneously judged as similar by a subject [72]–[78]).

To conclude, it should be stressed that the validity and generality of our conclusions are intrinsically limited by the limited extent of cortex that was explored through single-unit recordings here, as compared to the large cortical areas that are imaged in fMRI studies. In particular, single-unit recordings, unless paired with fMRI, cannot precisely target cortical regions that are known to represent specific object categories in monkey IT. For instance, our recordings did not specifically target any of the so-called monkey face patches [16], [31]–[33] or other IT regions that are rich of face selective neurons (summarized in [41], [42]) and, therefore, it is not surprising that no clusters entirely made of faces were found in our study (instead, the face cluster was part of larger clusters of objects with round shape and large area; e.g., see cluster # 4 in Fig. 5). In particular, given the across-monkey variability in the precise locations of face patches (Fig. 1 shows the range of possible locations for the three anterior face patches, based on [33]), the fact that we did not record from the dorsal bank of STS (thus excluding an overlap between our recording sites and face patch AF; see Fig. 1), and the fact that our recordings targeted the most medial part of IT (thus excluding any overlap between our recording sites and face patch AL; see Fig. 1), it is very unlikely that our IT sample contained a large fraction of face cells. Although an overlap between our recording sites and face patch AM is, in principle, possible (see Fig. 1), we verified that our sampled IT population did not contain any cell that was sharply tuned for faces, by computing, for each neuron, the Face Selectivity Index (FSI) proposed by [32]. Differently from what reported for face cells (e.g., see Fig. 2 in [32]), none of the neurons recorded in our study had a FSI exceeding 0.5 (see Fig. S3A). Moreover, those few cells with FSI∼0.5 typically did not show a sharp segregation between responses to faces and non-faces, and often had, as preferred stimuli, non-face objects (see Fig. S3B). Because of such a lack of sharp tuning for faces at the single cell level, it is not surprising that neither the *k*-means (see Fig. 4) nor the D-MST clustering algorithms (see Fig. 5) returned any pure cluster of faces. Very likely, if our recordings had targeted a wider extent of IT cortex (as in [14]) or had focused on sub-regions, within IT, that are rich of face-selective neurons [31]–[33], [41], [42], pure face clusters would have been observed. On the other hand, having found compact clusters of four-limbed animals and (to a lesser extent) birds suggests that at least a fraction of the neurons sampled in our study may have belonged to body selective IT regions (whose existence is also well-established in monkey IT [16], [31], [34]). In other words, our data, while showing that visual shape similarity is the main factor determining IT object representations, do not contradict the findings of earlier fMRI studies about the existence of face and body patches in IT.

In summary, the quantitative characterization of the IT response patterns performed in this study, while leaving open the possibility that a few, behaviorally salient semantic categories may be represented in monkey inferotemporal cortex, strongly reasserts the primary function of IT as a visual area, in which, in addition to moderately to highly complex shape information, a surprisingly large number of low-level visual properties is also represented.

## Materials and Methods

The data analyzed in this study were obtained from the same experiments described in [38]. We point the reader to this former study for a full description of surgical, behavioral, and recording procedures. Here we only provide those details that are essential to the understanding of the present study. All animal procedures were performed in accord with National Institute of Health guidelines and the Massachusetts Institute of Technology Committee on Animal Care.

### Visual stimuli and behavioral task

All recorded neurons were probed with a fixed set of 213 grayscale pictures of isolated objects that included: 1) 188 images of real-world objects belonging to 94 different categories (e.g., two hats, two accordions, two monkey faces, etc.) of the Caltech 101 database [79]; 2) five cars, five human faces, and five abstract silhouettes; 3) five patches of texture; 4) four low-contrast images of one of the objects; and 5) a blank frame. The full set is shown in Figure 2.

All objects subtended 2° of visual angle. During recordings, both monkeys were engaged in a simple recognition task that required the detection of a fixed target shape (a red triangle) that was presented at the end of a temporal sequence of object conditions drawn from our stimulus set (see [38]). The total number of stimulus conditions presented on each behavioral trial ranged from 3 to 20. The target was always the last in the sequence, and each monkey was rewarded for maintaining fixation (1.5° fixation window) until the appearance of the target and then making a saccade to a fixed visual field location (7° eccentricity) within 800 ms after the appearance of the target. Visual stimuli were presented at a rate of 5 per second; i.e., each stimulus condition was shown for 100 ms, followed by 100 ms of a gray screen (no stimulus), followed by another stimulus condition for 100 ms, etc. This task was meant to obtain a large amount of data, while still engaging the animal in a recognition task

### Neuronal recordings

During each recording session, a single extracellular metal electrode was advanced into IT through a stainless still guide tube that was inserted into a plastic cylindrical recording chamber (Crist Instruments). The chamber was placed over a craniotomy targeting the temporal lobe in the left hemisphere from the top of the skull. Over ∼6 months of daily recording sessions in the two monkeys, we sampled neurons over an ∼5×4 mm area of the ventral superior temporal sulcus and ventral surface lateral to the anterior middle temporal sulcus (Horsley-Clarke anteroposterior coordinates: 13–17 mm), corresponding to several 1 mm-spaced grid locations of the recording chamber (see Fig. 1). We recorded a total of 94 well-isolated single units. Each isolated neuron was initially tested for responsiveness across the set of 213 objects, presented at the center of gaze, using the following criterion: a neuron was considered responsive if its mean firing rate was significantly higher than background rate for at least one of these objects (*t* test, *p*<0.005). Responsive neurons were further screened to identify their preferred receptive field location (RF center) within a 2° span around the center of gaze (see [38] for details). Following these screening procedures, complete recordings from each neuron were obtained by presenting the full set of 213 objects at the neuron's RF center. Five to thirty presentation repetitions were collected for each object condition.

### Similarity metric for population responses

Neuronal responses were quantified by computing the average number of spikes per second fired by a neuron (i.e., average firing rate) across all repetitions of a given object, over a time window starting 100 ms and ending 200 ms after stimulus presentation. Similarly to what done in [14], the responses of a neuron across the object set were normalized by first subtracting their mean value (across the set) and then dividing by their standard deviation. This normalization compensated for differences in baseline activity and firing rate range across the recorded neuronal population, and allowed weighting equally all the neurons contributing to the population representation of a given object. Each visual object was thus represented by a neuronal population vector having as components the normalized responses of all the recorded neurons to that object. As in [14], the similarity between the population vectors representing two visual objects *i* and *j* was measured by computing their Pearson correlation coefficient (*r_ij_*). This metric was chosen because it is sensitive to the profile of activation of the neurons produced by a given object, rather than to the absolute magnitude of the activation. The distance (or dissimilarity) between the population vectors *i* and *j* was then defined as *d_ij_* = 1−*r_ij_* (the resulting dissimilarity matrix *D* is depicted in Fig. 3A).

### Unsupervised multivariate approaches

Three standard unsupervised approaches were used to understand the structure of visual object representations in IT: 1) average linkage hierarchical agglomerative clustering; 2) *k*-means clustering; and 3) Principal Components Analysis (PCA). The optimal number of *k*-means clusters was determined by the Bayes Information Criterion (BIC) and the Akaike Information Criterion (AIC) [52]. In addition to these standard approaches (whose description can be found in various textbooks and reviews; see [60], [61]), a more advanced method, developed within the domain of Statistical Physics, was also applied to strengthen our multivariate analysis: the D-MST clustering algorithm. This is a recently proposed method [53], [54], which allows interpolating between *partitional* clustering methods, such as *k*-means [60], [61] and Affinity Propagation [55], and hierarchical clustering methods [60], [61]. Its output is a so-called forest, i.e., a set of clusters, each of which is a tree (see Fig. 5). As the *k*-means, the D-MST clustering algorithm is non-deterministic, and takes two parameters as input: 1) the maximum depth of the trees *d*_max_ (i.e., the maximum number of links between any image and the image at the center of a tree); and 2) *λ*, which determines the number of resulting clusters (a bigger *λ* results in less clusters). As a way to determine the set of parameters that gave the most robust assessment of object clustering in IT, we imposed that both the number of clusters and their internal structure (i.e., the overlap between the clusters/trees resulting from repeated executions of the algorithm) be stable over a large range of parameters (50 executions of the algorithm were run for each assignment of the parameters). This yielded a single region of the parameter space fulfilling our stability criteria (see Fig. S2), corresponding to a partition of the object set into five trees with depth *d*_max_ = 6. From this region, five trees/clusters were extracted by keeping the most stable links across multiple runs of the D-MST (shown in Fig. 5). A more detailed description of the method is provided in Text S1.

### Clustering hypotheses

The neuronal-based object clusters produced by the algorithms described above were compared to object categories obtained according to three different clustering hypotheses: 1) shared semantic membership; 2) shared shape features; and 3) shared low-level visual properties.

Eleven semantic categories (shown in Fig. S1A) were built according to the criteria established in [14]. These categories were further grouped into the two superordinate categories of animate and inanimate objects.

Fifteen categories of objects sharing shape features (shown in Fig. S1B) were obtained as the result of object clustering in the output layer of a well-known hierarchical model of object recognition [43], [44], [68]. For our application, we have chosen the version of the model described in [44] (and downloaded from http://www.mit.edu/~jmutch/fhlib/ – version 8), which consists of four layers of artificial neural units named S1, C1, S2, and C2. Units S1 are a bank of Gabor filters with various orientations, spatial frequencies, positions and scales. Units C1 implement an OR-like operation on subsets of S1 afferent units, having the same orientation tuning but in different positions/scales. Units S2 perform a template matching (AND-like) operation on subsets of C1 afferent units to gain tuning for a particular combination of visual features. In this version of the model, the templates to which these units are tuned are random patches of images taken from the Caltech 101 database (different S2 units are built having as a template the same image patch, but at different positions and scales). In the output layer of the model, C2 units perform again an OR-like operation on subsets of S2 afferent units tuned for the same image patch, but at different positions and scales. In our instantiation of the models, 24,451 C2 output units were built. These units convey the more explicit (i.e., more shape selective and position/scale tolerant) representation of visual objects provided by the model. They could therefore be used to assess the similarity of our visual objects at the level of shared middle- to high-level shape features. This was achieved by running a *k*-means clustering algorithm over the representation of our object set provided by the model's output units, so as to obtain 15 groups of objects with similar features. The number of groups was set to 15 to match the optimal number of *k*-means clusters found in the IT neuronal representation using the BIC and AIC criteria (see previous section).

Eight categories of objects sharing low-level visual properties (shown in Fig. S1C) were defined on the base of four global properties of the images of the objects – luminance, contrast, area and aspect ratio. Each category contained 15 images having either the highest or the lowest values of one of such properties, which were defined as following. Luminance was defined as the average pixel intensity of the object image, divided by the maximum of the grayscale range (i.e., 255). Area was defined as the fraction of pixel, in the image frame, that was occupied by the image of the object. Note that object area, as defined here, is different from object size, which was fixed to ∼2° of visual angle for all the objects. Contrast was defined as: (median(pixels>128)−median(pixels<128))/(median(pixels>128)+median(pixels<128)). Aspect-ratio was defined as the maximum, across all the possible rotations, of the height of an object image divided by its width.

### Overlap score

For easier comparison with [14], the overlap between a *k*-means neuronal-based cluster and an object category was assessed with the same score used in that study, i.e., as the average of Ratio 1 and Ratio 2 (where Ratio 1 is the fraction of objects in the category overlapping with the cluster and Ratio 2 is the fraction of objects in the cluster overlapping with the category). Significance of the overlap score was assessed by a permutation test, in which, after reshuffling the objects among the clusters, the overlap scores were recomputed to obtain a null distribution. In the case of the clusters produced by the D-MST algorithm, the overlap score was defined as the intersection between a given cluster and a given category, divided by their union. Significance was assessed by a permutation test, which, as explained in the Results, took into account the presence of twin exemplars in the object set (see Text S1 for a full description).

### Fisher Linear Discriminant (FLD) analysis

The ability of the recorded IT population to code the category membership of visual objects was estimated by building binary Fisher Linear Discriminants/classifiers (FLDs). Each classifier was trained to find the best hyperplane separating, in the neuronal representation space, the objects belonging to a given category from all other objects (FLDs achieve this by maximizing the ratio of the between-category variance to the within-category variance [60]). Since the neurons were not recorded simultaneously, pseudo-population response vectors were built by assigning to each component of any given vector the number of spikes that each neuron fired in a randomly sampled (with replacement) presentation of a given object. Seven of such pseudo-population vectors were built for each object (being seven the median number of repetitions per object and neuron obtained during recordings). The entire set of pseudo-population vectors were built anew for each cross-validation run of the classifier (see below).

Classifier performance was measured in cross-validation loops. In each loop, the classifier was trained using all the available population vectors, with the exception of all the vectors corresponding to two left-out objects, one from the category that the classifier was being trained to discriminate, and one from the complementary set. For any given classification task, performance at correctly classifying left-out vectors was measured over a set of 30 cross-validation loops, with a different pair of left-out objects randomly chosen in each loop. Each set of cross-validation loops constituted a cross-validation run and 3,500 such runs were executed for each binary classification task, so as to obtain average performances and their standard errors (see histogram bars and their error bars in Fig. 6). Following [67], significance of the classification performance was assessed with a permutation test, in which object labels were shuffled before executing each cross-validation loop, so as to obtain null distributions of the performance (see gray circles and their error bars in Fig. 6).

The same cross-validation scheme was used to test the significance of the performance at classifying “pruned” categories, i.e., object sets obtained by sub-sampling the original categories and their complementary (negative) sets, so as to disentangle as much as possible semantic from visual information. Pruned categories were built by solving two constraint optimization problems. The goal was to build the largest possible set of objects belonging to a given category, such that visual features would not interfere with semantic features and vice versa. Therefore, when testing for discrimination of a semantic category, we imposed that no pair of objects would belong to the same shape-based or low-level category; when testing for discrimination of a shape-based or low-level category, we imposed that no pair of objects would belong to the same semantic category. For example, when testing for discrimination of the *round* category, which includes, among other objects, also many faces (see Fig. S1B), only one of the faces was allowed to be included in the pruned category (see second panel in Fig. 6B). We also imposed that no twins appeared together in any pruned category. These constraints applied both to the positive and to the negative (i.e., complementary) classes, and, therefore, the problem had to be solved twice every time. This problem can be easily framed as an integer linear programming problem, and solved using standard kits (http://www.gnu.org/software/glpk/). Since several solutions are possible, we introduced a small random noise and solved the problem repeatedly in order to sample from the set of all solutions – one pruned version of the category to be discriminated (and the complementary object set) was built for each of the 3,500 cross-validation runs (see above). In a few instances (e.g., the *fishes*), the resulting “pruned” category had too few objects for the linear classifier analysis to be performed. To assess the significance of the classification performances we built null categories, by first shuffling the twin indices over the whole stimulus set, and then sampling the null positive and negative categories with the same constraints as above. That is, we required the null “semantic” categories to be made of visually dissimilar objects, and the null “visual-based/low-level” categories to be made of objects with different semantic membership. In addition, we forced the null categories to have the same size as the corresponding pruned categories.

## Supporting Information

Figure S1 **Object categories of the three clustering hypotheses.** The 11 semantic categories (A), the 15 shape-based categories (B) and the 8 low-level object categories (C). See main text (Materials and Methods) for a definition of the categories.
(TIF)

Figure S2 **Computation of the stability region in the parameter space of the D-MST clustering algorithm.** Average number of clusters and average overlap (inset) in repeated D-MST clustering outcomes, showing the only stable region of the parameters (found at *d*_max_ = 6, λ ∈ [0.74,0.88]). The main panel shows the average number of clusters at *d*_max_ = 6 as a function of the parameter λ (error bars = standard deviations across 50 repeated outcomes of D-MST clustering). The stable region is highlighted in light red. The yellow line represents the linear fit for that region, corresponding to a number of clusters = 4.55±0.03. The inset shows the average overlap between repeated outcomes of the clustering at *d*_max_ = 6 as a function of λ. For each point, the average overlap is computed over all D-MST outcomes in a sliding window of width 0.15 centered at that point. The blue dot represents the value corresponding to the stable region (overlap = 0.94±0.04). The span of that region is highlighted in light green.
(TIF)

Figure S3 **Face selectivity of the recorded inferotemporal neurons.** (A) Histogram showing the distribution of the Face Selectivity Index (FSI) across the recorded population of IT neurons. The index was defined, according to Tsao et al (*Science*, 2006), as: FSI = (mean response_faces_−mean response_non-face objects_)/(mean response_faces_+mean response_non-face objects_). Differently from Tsao et al, no neurons were found with a sharp tuning for faces (i.e., with FSI larger than 0.5). (B) Rank-order tuning curves for the four neurons with the largest FSI. Each plot shows the response (i.e., average firing rate) of a neuron across the set of 213 objects used in our study (shown in Fig. 2). For each neuron, objects along the abscissa are ranked based on the response they evoked. The responses evoked by faces (either human, monkey, or dog faces) are marked by specific symbols (see legend in the figure). These tuning curves show how, even for our most face selective cells, non-face objects were often the cells' preferred stimuli, and no sharp segregation between responses to faces and non-face objects was found.
(TIF)

Text S1 **Supporting** **Materials and Methods** **.** Description of how the D-MST clustering algorithm was applied in the context of this study.
(DOC)
