# Supplementary figures and images for: Shape Similarity, Better than Semantic Membership, Accounts for the Structure of Visual Object Representations in a Population of Monkey Inferotemporal Neurons
Source: PLoS Comput Biol. 2013 Aug 8;9(8):e1003167. doi: 10.1371/journal.pcbi.1003167 (PMC3738466; doi:10.1371/journal.pcbi.1003167)

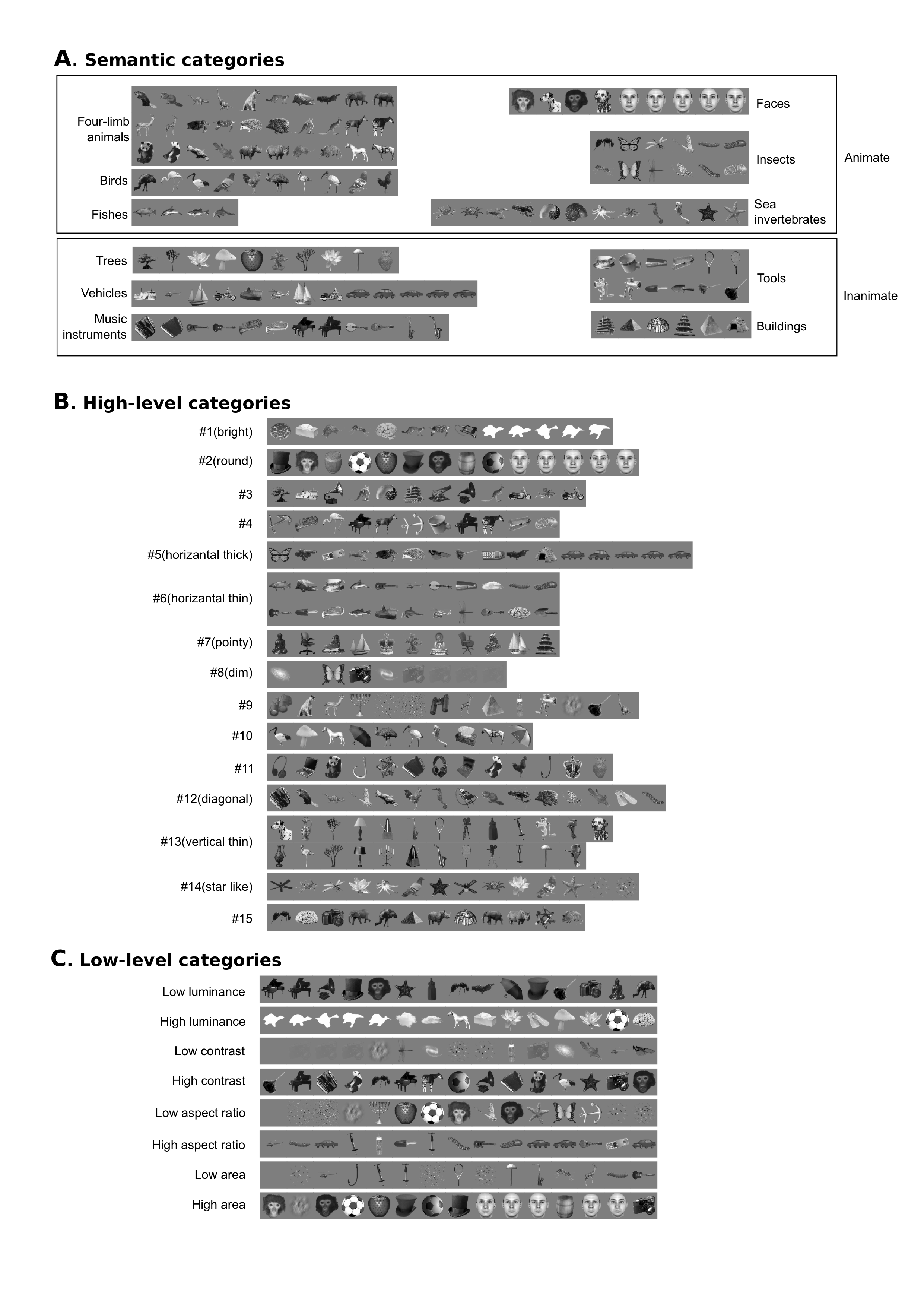

Supplement: Figure S1 — Object categories of the three clustering hypotheses. The 11 semantic categories (A), the 15 shape-based categories (B) and the 8 low-level object categories (C). See main text (Materials and Methods) for a definition of the categories. (TIF) [file pcbi.1003167.s001.tif]

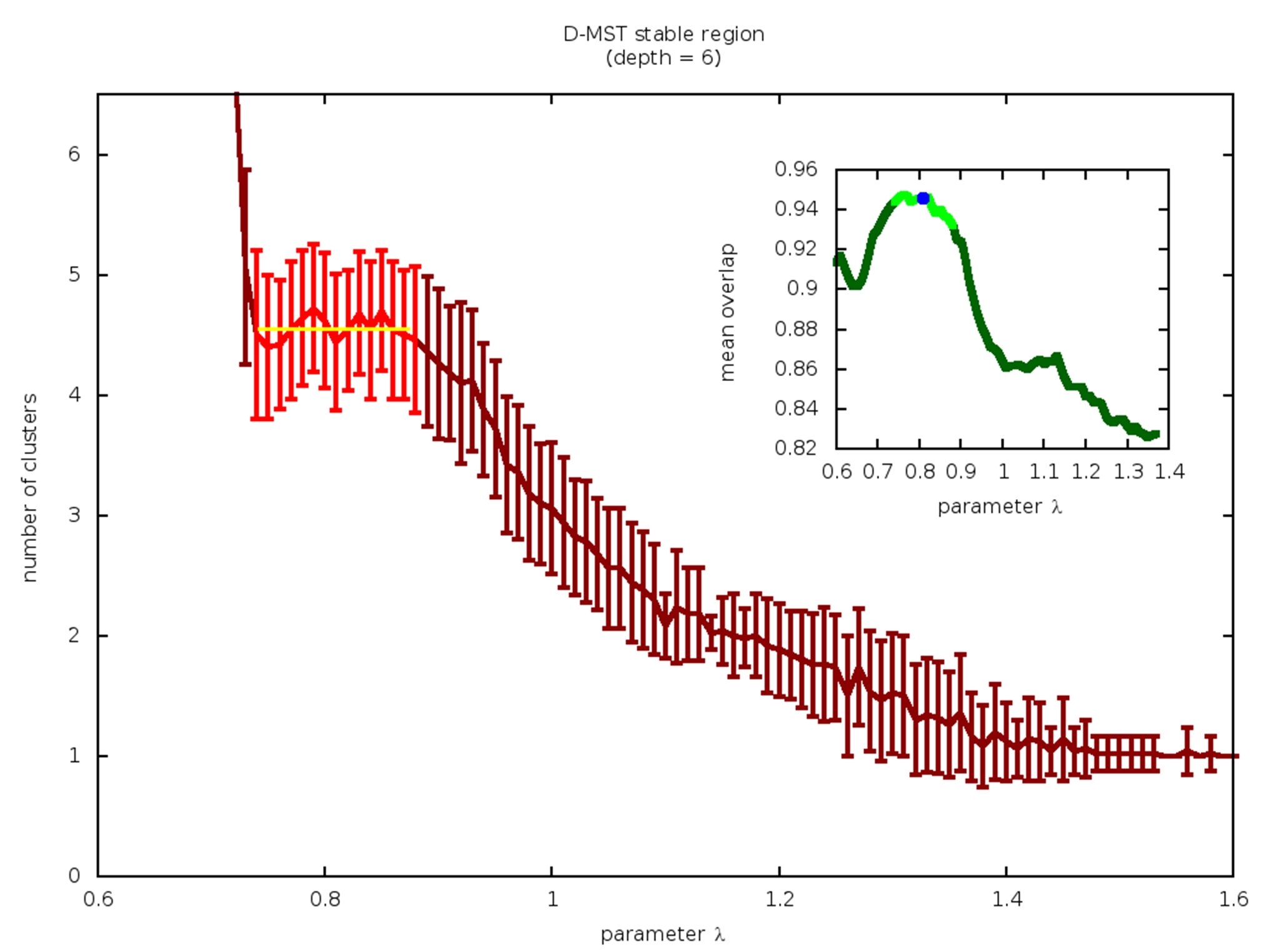

Supplement: Figure S2 — Computation of the stability region in the parameter space of the D-MST clustering algorithm. Average number of clusters and average overlap (inset) in repeated D-MST clustering outcomes, showing the only stable region of the parameters (found at d max = 6, λ ∈ [0.74,0.88]). The main panel shows the average number of clusters at d max = 6 as a function of the parameter λ (error bars = standard deviations across 50 repeated outcomes of D-MST clustering). The stable region is highlighted in light red. The yellow line represents the linear fit for that region, corresponding to a number of clusters = 4.55±0.03. The inset shows the average overlap between repeated outcomes of the clustering at d max = 6 as a function of λ. For each point, the average overlap is computed over all D-MST outcomes in a sliding window of width 0.15 centered at that point. The blue dot represents the value corresponding to the stable region (overlap = 0.94±0.04). The span of that region is highlighted in light green. (TIF) [file pcbi.1003167.s002.tif]

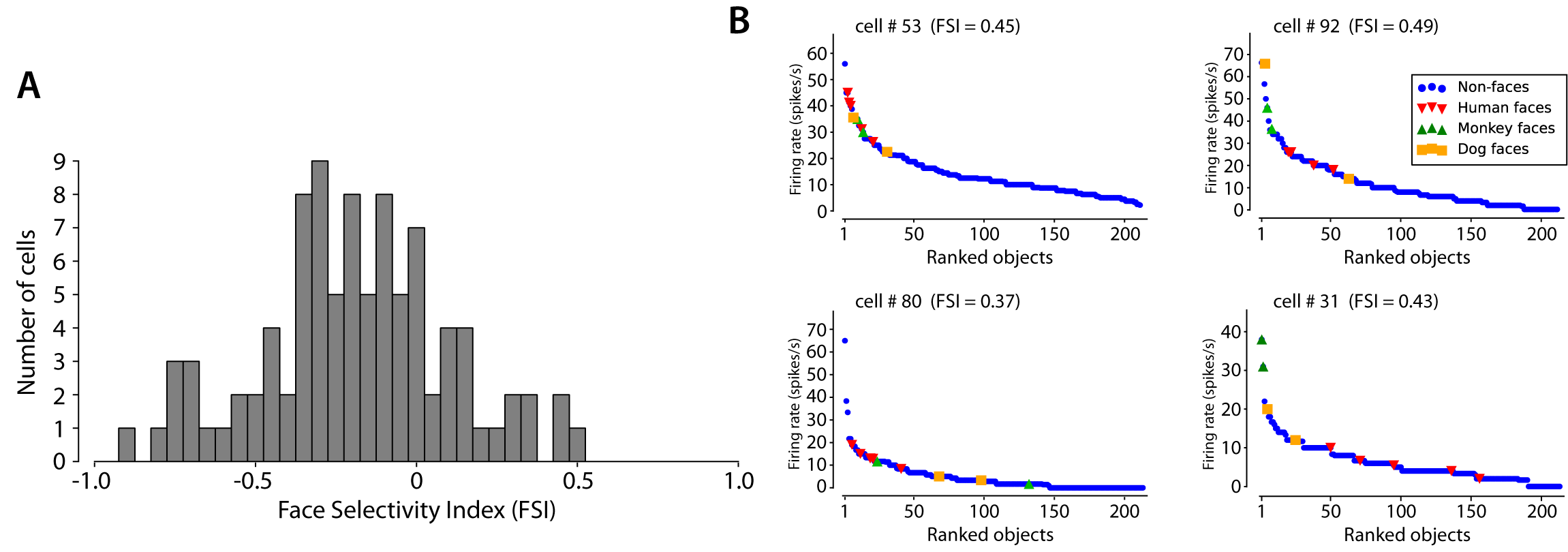

Supplement: Figure S3 — Face selectivity of the recorded inferotemporal neurons. (A) Histogram showing the distribution of the Face Selectivity Index (FSI) across the recorded population of IT neurons. The index was defined, according to Tsao et al (Science, 2006), as: FSI = (mean responsefaces−mean responsenon-face objects)/(mean responsefaces+mean responsenon-face objects). Differently from Tsao et al, no neurons were found with a sharp tuning for faces (i.e., with FSI larger than 0.5). (B) Rank-order tuning curves for the four neurons with the largest FSI. Each plot shows the response (i.e., average firing rate) of a neuron across the set of 213 objects used in our study (shown in Fig. 2). For each neuron, objects along the abscissa are ranked based on the response they evoked. The responses evoked by faces (either human, monkey, or dog faces) are marked by specific symbols (see legend in the figure). These tuning curves show how, even for our most face selective cells, non-face objects were often the cells' preferred stimuli, and no sharp segregation between responses to faces and non-face objects was found. (TIF) [file pcbi.1003167.s003.tif]
