# Supplementary material for: Shape Similarity, Better than Semantic Membership, Accounts for the Structure of Visual Object Representations in a Population of Monkey Inferotemporal Neurons
Source: PLoS Comput Biol. 2013 Aug 8;9(8):e1003167. doi: 10.1371/journal.pcbi.1003167 (PMC3738466; doi:10.1371/journal.pcbi.1003167)
Supplement: Text S1 — Supporting Materials and Methods . Description of how the D-MST clustering algorithm was applied in the context of this study. (DOC) [file pcbi.1003167.s004.doc]

**Shape similarity, better than semantic membership, accounts for the structure of visual object representations in a population of monkey inferotemporal neurons**

Carlo Baldassi^1,2,*^, Alireza Alemi-Neissi^2,3,*^, Marino Pagan^4,5,*^, James J DiCarlo^4^, Riccardo Zecchina^1,2^, Davide Zoccolan^3,4^

1. Department of Applied Science and Technology & Center for Computational Sciences, Politecnico di Torino, 10129 Torino, Italy

2. Human Genetics Foundation (HuGeF), Torino, 10126 Torino, Italy

3. International School for Advanced Studies (SISSA), Trieste, 34136, Italy

4. Department of Brain and Cognitive Sciences and McGovern Institute for Brain Research, Massachusetts Institute of Technology (MIT), Cambridge, MA 02139, USA

5. Department of Psychology, University of Pennsylvania, Philadelphia, PA 19104, USA

^*^ CB, AAN and MP contributed equally to this work

**Text S1**

## The D-MST clustering algorithm

As explained in Materials and Methods, the D-MST algorithm takes two parameters as input, *λ* and *d*_max_, and outputs a forest of trees as a result. The first parameter, *λ*, takes real positive values and controls the number of trees (i.e., clusters) in the outcome (a bigger *λ* results in a smaller number of clusters), while the second parameter, *d*_max_, is an integer greater or equal than 2 and determines the maximum depth of the resulting trees, as described in the next section.

### *The role of d_max_ and the relation between D-MST, Affinity Propagation and Single-linkage hierarchical clustering*

The D-MST algorithm can interpolate between Affinity Propagation [1], which returns a partitioning of the input data (like *k*-means) and Single-linkage hierarchical clustering [2], which returns instead a hierarchical organization of the data, by properly setting the parameter *d*_max_. When *d*_max_= 2, each tree has a central element (the so-called root) and all other elements in the tree are linked to it, and the D-MST algorithm becomes exactly equivalent to Affinity Propagation and essentially equivalent to *k*-means (the resulting clustering is good for roughly spherical clusters, because that is the underlying assumption). When *d*_max_ is bigger than 2, elements can be linked to other elements different from the root, in such a way that the maximum number of links between an element and the root is given by *d*_max_-1. By letting *d*_max_ be greater or equal than the number of elements in the input (and by taking a large enough *λ*), the output is a single tree of (effectively) unbounded depth, and therefore the D-MST algorithm reproduces the Minimum Spanning Tree algorithm in that regime (the clustering information is fully contained in the hierarchical structure, without partitioning, as in the Single-linkage hierarchical clustering approach).

### *Optimal choice of parameters for the D-MST method*

The region of the D-MST parameter space we explored had $\lambda_{\text{max}}$ ranging from 2 to 9 and $\lambda$ ranging from 0 to $\lambda_{\text{max}}$, where $\lambda_{\text{max}}$ is the value after which the outcome always forms a single cluster (this value is different for each value of $d_{\text{max}}$, and decreases as $d_{\text{max}}$ increases). The $\lambda$ range step-size was chosen to be small enough to allow the detection of relevant plateaus in the plot of the average number of clusters vs. $\lambda$ (with fixed $d_{\text{max}}$; see Fig. S2). Namely, the step-size was 0.05 for $d_{\text{max}}$ from 2 to 4 and 0.01 for $d_{\text{max}}$ from 5 to 9.

In order to identify the stable regions of the parameter space, we used a two-fold criterion, requiring 1) the average number of clusters to be stable across a large enough range of the parameter $\lambda$ (for a given value of $d_{\text{max}}$), and 2) the average overlap between different solutions in the considered region to be large enough.

Intuitively, the criterion can be interpreted as such. Since the effect of increasing *λ* is that the clusters which are close to one another tend to merge into larger clusters, the fact that the number of clusters is stable when varying *λ* suggests that the resulting clusters are well separated. However, since the algorithm is heuristic and we consider multiple runs (not only because we vary *λ*, but also because we take 50 samples for each choice of the parameters), the sheer number of clusters is not a sufficient condition, because the same number of clusters could arise from very different outcomes of the algorithm, and thus we also need to make sure that the individual links are stable enough in the D-MST outcome, by requiring that the overlap between solutions is high enough. Note that this last condition alone does not guarantee that the number of clusters is stable, since the number of clusters is a quantity that can be very sensitive to the presence or absence of a single link, while the overlap is an average over all the links.

The stability of the number of clusters was evaluated by fitting the cluster number vs. *λ* plot with a constant function, using every possible range of *λ* values greater than 5% of$\lambda_{\text{max}}$, and requiring the relative fit error to be lower than 1% (see the plateau in Fig. S2).

The overlap between different outcomes of D-MST was defined like this: use *a* and *b* to denote the two outcomes, and for any outcome *s* we say $x_{\text{ij}}^{s}$ is equal to 1 if the edge *(i,j)* exists in outcome *s* and zero otherwise (we neglect the direction of the edges within the D-MST solution); then the overlap *q(a,b)* is:

(eq. 3)

It can be easily verified that *q(a,b)* is between 0 and 1, and it is 1 if and only if the two graphs are equal.

For any given range of values of *λ,* we computed the average overlap among any pair of D-MST outcomes, and required the average to be greater than 0.9 for the clustering to be considered stable.

As shown in Figure S2, the only stable region which we found according to these criteria had $d_{\text{max}}=6$ and ( for $d_{\text{max}}=6$). The plateau fit for this region yielded $4.55\pm0.03$ clusters, and the average overlap among the 750 outcomes in this region was 0.94 (with a standard deviation of 0.04).

### *Computation of the final outcome: the most stable D-MST clusters*

As described in the previous section, we isolated a single region of the parameter space that fulfilled our stability criterion. Considering such region, then, we extracted, for every pair of objects *(i,j)*, the frequency $\nu_{\text{ij}}$ with which the edge connecting them appeared in the outcome of all the individual D-MST runs (i.e. 50 runs for each value of *λ*, times the number of *λ* values within the considered region). Finally, to obtain a definite clustering of the data, we used the $\nu_{\text{ij}}$ frequencies thus obtained to build a new forest, in this way: we started with an empty graph (each object forming a cluster by its own), and progressively merged the clusters by adding links in the order given by the $\nu_{\text{ij}}$ frequencies, but avoiding the ones which would introduce loops in the graph, until the number of clusters matched the stable one obtained on average from the D-MST runs.

### *Overlap score and statistical test*

As a measure of the significance of the object categories belonging to our three representation hypotheses in the outcome of the D-MST algorithm we used an overlap-based score, and evaluated its statistical significance by a permutation test.

The overlap was defined in such a way as to account for both the partitioning of objects into clusters and the internal unrooted-tree structure of the clusters themselves, as follows. For each category *c* and each D-MST cluster/tree *t*, we computed the intersection *i = c* ∩ *t* between *c* and the set of elements in *t*. Then we computed a set *s* as the set of elements of the connected component in *t* (i.e., a contiguous sub-tree of *t*) induced by *i*, i.e., obtained by taking the union of all paths along *t* between any pair of elements of *i* (note that with this definition *c* ∩ *s = i*). Finally, we computed the overlap score as the ratio between the sizes of the intersection and the union of *c* and *s*: *o = |c* ∩ *s | / |c* ∪ *s|*. To each category *c* we associated the cluster which gives the maximum score *o*.

The significance of the overlap score was assessed by comparing the value *o* obtained as described above with the distribution of the scores for random categories of the same size as the one under scrutiny. The fact that the score uses the internal structure of the data under consideration imposes a careful choice of the null hypothesis. If the presence of twins (i.e., multiple, very similar exemplars of the same objects) in the dataset is not taken into account, the statistical test will give a strong bias towards any category which is mostly composed of groups of twins, since most of the twins tend to be closely associated in the neural representation (see Fig. 5). Therefore, the random categories that we used for the permutation test were constructed in the following way: we extracted random groups of twins until the size of the resulting set was equal to that of the category under consideration (discarding elements in excess).

**Supplemental References**

1. Frey BJ, Dueck D (2007) Clustering by Passing Messages Between Data Points. Science 315: 972–976. doi:10.1126/science.1136800.

2. Duda RO, Hart PE, Stork DG (2001) Pattern classification. Wiley. 688 p.
